# Synthesis, Anti-tubulin and Antiproliferative SAR of Steroidomimetic Dihydroisoquinolinones

**DOI:** 10.1002/cmdc.201400017

**Published:** 2014-03-05

**Authors:** Mathew P Leese, Fabrice L Jourdan, Meriel R Major, Wolfgang Dohle, Mark P Thomas, Ernest Hamel, Eric Ferrandis, Mary F Mahon, Simon P Newman, Atul Purohit, Barry V L Potter

**Affiliations:** [a] Medicinal Chemistry, Department of Pharmacy & Pharmacology, University of BathBath, BA2 7AY (UK) E-mail: B.V.L.Potter@bath.ac.uk; [b] Screening Technologies Branches, National Cancer InstituteFrederick, MD 21702 (USA); [c] Institut de Recherche Henri Beaufour91966 Les Ulis Cedex (France); [d] X-ray Crystallographic Suite, Department of Chemistry, University of BathBath, BA2 7AY (UK); [e] Oncology Drug Discovery Group, Section of Investigative Medicine, Hammersmith Hospital, Imperial College LondonLondon, W12 0NN (UK)

**Keywords:** colchicine, dihydroisoquinolinones, electrostatic repulsion, microtubules, tubulin

## Abstract

A SAR translation strategy adopted for the discovery of tetrahydroisoquinolinone (THIQ)-based steroidomimetic microtubule disruptors has been extended to dihydroisoquinolinone (DHIQ)-based compounds. A steroid A,B-ring-mimicking DHIQ core was connected to methoxyaryl D-ring mimics through methylene, carbonyl, and sulfonyl linkers, and the resulting compounds were evaluated against two cancer cell lines. The carbonyl-linked DHIQs in particular exhibit significant in vitro antiproliferative activities (e.g., 6-hydroxy-7-methoxy-2-(3,4,5-trimethoxybenzoyl)-3,4-dihydroisoquinolin-1(2*H*)-one (**16 g**): GI_50_ 51 nm in DU-145 cells). The broad anticancer activity of DHIQ **16 g** was confirmed in the NCI 60-cell line assay giving a mean activity of 33 nm. Furthermore, 6-hydroxy-2-(3,5-dimethoxybenzoyl)-7-methoxy-3,4-dihydroisoquinolin-1(2*H*)-one (**16 f**) and **16 g** and their sulfamate derivatives **17 f** and **17 g** (2-(3,5-dimethoxybenzoyl)-7-methoxy-6-sulfamoyloxy-3,4-dihydroisoquinolin-1(2*H*)-one and 7-methoxy-2-(3,4,5-trimethoxybenzoyl)-6-sulfamoyloxy-3,4-dihydroisoquinolin-1(2H)-one, respectively) show excellent activity against the polymerization of tubulin, close to that of the clinical combretastatin A-4, and bind competitively at the colchicine binding site of tubulin. Compounds **16 f** and **17 f** were also shown to demonstrate in vitro anti-angiogenic activity. Additionally, X-ray and computational analyses of **17 f** reveal that electrostatic repulsion between the two adjacent carbonyl groups, through conformational biasing, dictates the adoption of a “steroid-like” conformation that may partially explain the excellent in vitro activities.

## Introduction

**Figure 1:**
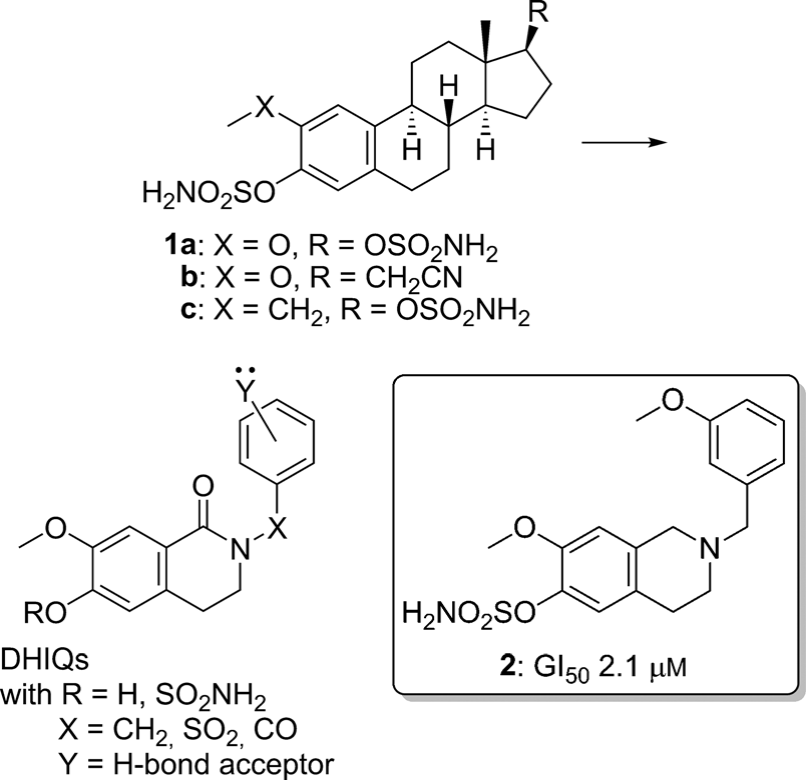
Design of DHIQ-based steroidomimetic microtubule disruptors.

In previous studies we optimised sulfamoylated estratrienes **1 a**–**b** (Figure 1) as anticancer agents.[1–6] These compounds exhibit antiproliferative activity against a range of human cancer cell lines and are also capable of inhibiting angiogenesis. This dual mechanism of action can be ascribed to their ability to inhibit normal microtubule dynamics and, in addition to good oral bioavailability and excellent in vivo activity, they proved capable of inhibiting the growth of cell lines resistant to existing microtubule disruptors such as the taxanes. To develop further series of compounds that share this mechanism of action we were drawn to investigate whether, by translating key pharmacophoric elements from the steroidal series into nonsteroidal motifs, we could generate new microtubule disruptors with further enhanced activity and/or physicochemical properties. In initial studies[7, 8] we used a tetrahydroisoquinoline (THIQ) decorated at C6 and C7 to mimic the steroidal A,B-ring of the estratrienes tethered through N2 to a D-ring mimic, initially a benzyl group, which projects a hydrogen bond acceptor into the appropriate region of space to address the pharmacophore for antiproliferative activity in that region. This delivered a series of microtubule disruptors with antiproliferative activity in the micromolar range that could be further optimised by introducing a substituent at C3 to sterically inhibit the free rotation of the *N*-benzyl group and thus favour conformational populations in which the *N*-benzyl group mimics the steroidal D-ring. In this manner compounds displaying nanomolar activity (equivalent to that of the steroid derivatives upon which their design was based) were elaborated.[9] In tandem, chimeric microtubule disruptors built from the THIQ core and the trimethoxy aryl motif common to many colchicine site binders were constructed.[10, 11]

In the present work we sought to develop new microtubule disruptors through integration of lessons learned in work on the estratrienes and the THIQ-based systems. By using an alternate heterocyclic motif, the dihydroisoquinolinone (DHIQ) to mimic the steroidal A,B-ring system, we envisaged that some conformational pre-organisation could be achieved through electrostatic repulsion. Tethering the D-ring-mimicking aryl group through a carbonyl linkage, repulsion of the two carbonyl groups of the imide should cause adoption of a dihedral angle in which they minimise their electrostatic clash; this should deliver a conformation in which the D-ring mimic projects into a region of space broadly analogous to a steroidal D-ring, thus decreasing rotation to achieve optimal binding. To assess this strategy a series of 2′-, 3′- and 4′-methoxybenzoyl DHIQs was synthesised alongside corresponding *N*-benzyl and *N*-arylsulfonyl DHIQ series for which no positive conformational biasing is envisaged (Figure 1).

## Results and Discussion

### Chemistry

**Scheme 1:**
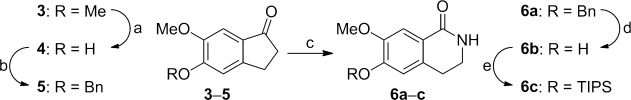
Synthesis of the DHIQ core structure. *Reagents and conditions:* a) piperidine/H_2_O, 140 °C; b) BnBr, K_2_CO_3_, DMF, RT; c) NaN_3_, CH_3_SO_3_H, CH_2_Cl_2_, 0 °C→RT; d) H_2_, Pd/C, THF/MeOH, RT; e) TIPSCl, imidazole, CH_2_Cl_2_, RT.

The synthetic strategy to access the dihydroisoquinolinone core structure is outlined below (Scheme 1). 5,6-Dimethoxyindan-1-one **3** was selectively demethylated in aqueous piperidine and the product subsequently reacted with benzyl bromide to furnish compound **5** in good yield. Reaction of **5** with sodium azide and methanesulfonic acid in dichloromethane gave dihydroisoquinolinone **6 a**. Some of the material was transformed into the corresponding unprotected phenol **6 b**, by treatment with hydrogen and palladium on charcoal, that was subsequently protected with TIPSCl in dichloromethane to afford compound **6 c** (Scheme 1).

**Scheme 2:**
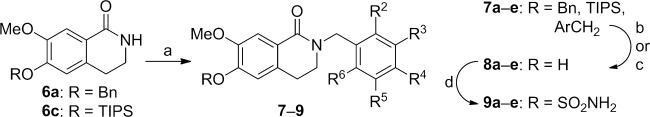
Synthesis of functionalised *N*-benzyl-substituted DHIQs. *Reagents and conditions:* a) NaH, 0 °C, DMF, then benzyl halide, RT; b) H_2_, Pd/C, THF/MeOH, RT; c) TBAF, THF, RT; d) H_2_NSO_2_Cl, DMA, RT.

*N*-Benzylation was then performed on compound **6 c**. Under the conditions applied in these reactions TIPS-deprotection and subsequent benzylation of the unprotected phenol was also observed to give compounds **7 a**–**c**. In two further experiments the more stable benzyl-protected compound **6 a** was reacted in the same manner to afford compounds **7 d**–**e**. These were transformed into the corresponding unprotected phenols **8 a**–**e** either by treatment with hydrogen and Pd/C or with TBAF (only for **7 b**) in good yields. Treatment of **8 a**–**e** with sulfamoyl chloride in DMA[12] gave the corresponding sulfamates **9 a**–**e** (Scheme 2).

**Scheme 3:**
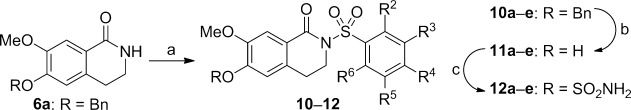
Synthesis of functionalised *N*-arylsulfonyl-substituted DHIQs. *Reagents and conditions:* a) NaH, DMF, 50 °C, then ArSO_2_Cl, DMF, RT or 40 °C; b) H_2_, Pd/C, THF/EtOH, RT; c) H_2_NSO_2_Cl, DMA, RT.

A second compound set was produced by *N*-sulfonylating **6 a** with various arylsulfonyl chlorides. Compounds **10 a**–**e** were transformed into the corresponding unprotected phenols **11 a**–**e**, by treatment with hydrogen and Pd/C, that were then reacted with sulfamoyl chloride in DMA to afford the corresponding sulfamates **12 a**–**e** (Scheme 3).

**Scheme 4:**
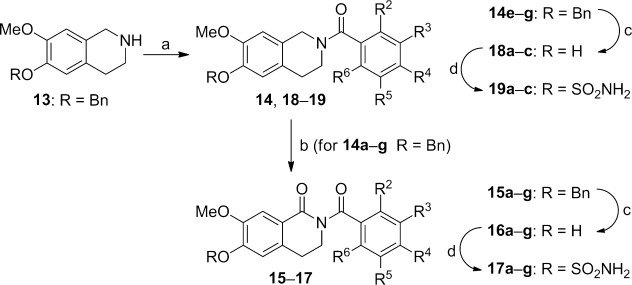
Synthesis of functionalised *N*-benzoyl-substituted DHIQs and THIQs. *Reagents and conditions:* a) ArCOCl, Et_3_N, CH_2_Cl_2_, RT; b) KMnO_4_, 18-crown-6, CHCl_3_, RT; c) H_2_, Pd/C, THF/MeOH, RT; d) H_2_NSO_2_Cl, DMA, RT.

For the synthesis of *N*-benzoylated compounds a direct strategy starting from **6 a** proved not very successful. Therefore, an alternate approach using benzyl-protected THIQ **13**[8] as starting material was adopted. *N*-Benzoylation using various benzoyl chlorides followed by permanganate oxidation gave access to the desired *N*-benzoylated dihydroisoquinolines **15 a**–**g**. These were reacted with hydrogen and Pd/C and the product treated with sulfamoyl chloride in DMA to give sulfamates **17 a**–**g**. Additionally, a set of control compounds without the carbonyl at C1 was achieved by hydrogenation of compounds **14 e**–**g** with Pd/C to give phenols **18 a**–**c**. Treatment with sulfamoyl chloride in DMA then afforded sulfamates **19 a**–**c** (Scheme 4).[8]

### Biology

The compounds were then evaluated in vitro for their ability to inhibit the proliferation of DU-145 human prostate cancer cells and MDA MB-231 breast cancer cells. As can be observed in Table 1, the *N*-benzyl derivatives, regardless of the nature of substitution of the benzyl motif, prove to be uniformly inactive in contrast to the corresponding THIQ series in which the 3′-methoxy compound **2** (Figure 1) exhibits a GI_50_ value of 2.1 μm.[7, 8] The addition of a carbonyl at C1 in this case is clearly detrimental, and it seems reasonable to suggest that this group, through steric considerations, disfavours adoption of a conformation in which the benzyl group can potentially mimic the steroid D-ring. Likewise, the series of *N*-arylsulfonyl derivatives prove to be inactive. The corresponding series of *N*-arylsulfonyl THIQs also fails to display significant activity, a fact that we ascribe to the likely unacceptable steric bulk of the sulfonyl group at the site of binding.[8] The *N*-benzoyl DHIQs, in contrast, provide interesting activity in both the 6-hydroxy and 6-sulfamoyloxy series. Here, both the 2′- and 3′-methoxy compounds give low micromolar GI_50_ values against the proliferation of both DU-145 and MDA MB-231 cells. It is notable that the 6-hydroxy compounds **16 a**–**b** display superior activity to the 6-*O*-sulfamates **17 a**–**b** and that, as found for the *N*-benzyl THIQs (with which we believed they should act in an analogous manner), the 3′-substituted derivative displays the highest activity. In contrast, as found in the *N*-benzyl THIQ series, the 4′-substituted compounds **16 d** and **17 d** are inactive. Several polymethoxylated compounds were also evaluated revealing that, although 4′-substitution alone did not afford activity, it is tolerated as the 3′,4′-dimethoxy compounds **16 e** and **17 e** display good activity and are more active than the 3′-methoxy derivatives **16 b** and **17 b**. The major step forward in activity was realised when the 3′,5′-dimethoxy derivative **16 f** and its sulfamate **17 f** were evaluated, with roughly equivalent activity (GI_50_ values of 215 and 179 nm for DU-145, respectively). Compounds **16 f** and **17 f** are >10-fold more active than the 3′-methoxy derivatives **16 b** and **17 b**. We postulate that this symmetric substitution adds a further entropic advantage in combination with the pre-organisation we believed would derive from the repulsion of the carbonyls of the imide discussed above. In any case, the activity of **16 f** and **17 f** is equal to that of both the conformationally biased THIQs and the steroidal derivatives from which the nonsteroidal design was conceived.

**Table 1:** Antiproliferative activity of DHIQs against DU-145 human prostate cancer cells and MDA MB-231 human breast cancer cells in vitro

Compd	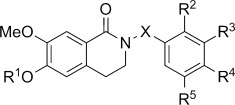						GI_50_ [μm]^[a]^	
	R^1^	X	R^2^	R^3^	R^4^	R^5^	DU-145	MDA MB-231
**8 a**	H	CH_2_	OMe	H	H	H	>100	ND^[b]^
**9 a**	SO_2_NH_2_	CH_2_	OMe	H	H	H	>100	ND^[b]^
**8 b**	H	CH_2_	H	OMe	H	H	>100	ND^[b]^
**9 b**	SO_2_NH_2_	CH_2_	H	OMe	H	H	>100	ND^[b]^
**8 c**	H	CH_2_	H	H	OMe	H	>100	ND^[b]^
**9 c**	SO_2_NH_2_	CH_2_	H	H	OMe	H	>100	ND^[b]^
**7 d**	Bn	CH_2_	H	OMe	H	OMe	>100	ND^[b]^
**8 d**	H	CH_2_	H	OMe	H	OMe	>100	ND^[b]^
**9 d**	SO_2_NH_2_	CH_2_	H	OMe	H	OMe	>100	ND^[b]^
**7 e**	Bn	CH_2_	H	OMe	OMe	OMe	>100	ND^[b]^
**8 e**	H	CH_2_	H	OMe	OMe	OMe	>100	ND^[b]^
**9 e**	SO_2_NH_2_	CH_2_	H	OMe	OMe	OMe	>100	ND^[b]^
**10 a**	Bn	SO_2_	OMe	H	H	H	>100	>100
**11 a**	H	SO_2_	OMe	H	H	H	>100	13.4
**12 a**	SO_2_NH_2_	SO_2_	OMe	H	H	H	>100	>100
**10 b**	Bn	SO_2_	H	OMe	H	H	>100	>100
**11 b**	H	SO_2_	H	OMe	H	H	>100	>100
**12 b**	SO_2_NH_2_	SO_2_	H	OMe	H	H	>100	>100
**11 c**	H	SO_2_	H	H	OMe	H	>100	>100
**12 c**	SO_2_NH_2_	SO_2_	H	H	OMe	H	>100	>100
**10 d**	Bn	SO_2_	H	Cl	H	H	>100	>100
**11 d**	H	SO_2_	H	Cl	H	H	>100	>100
**12 d**	SO_2_NH_2_	SO_2_	H	Cl	H	H	>100	>100
**11 e**	H	SO_2_	CO_2_Me	H	H	H	>100	>100
**12 e**	SO_2_NH_2_	SO_2_	CO_2_Me	H	H	H	>100	13
**15 a**	Bn	CO	OMe	H	H	H	>100	43.6
**16 a**	H	CO	OMe	H	H	H	6.42	5.6
**17 a**	SO_2_NH_2_	CO	OMe	H	H	H	9.1	7.1
**16 b**	H	CO	H	OMe	H	H	3.63	1.89
**17 b**	SO_2_NH_2_	CO	H	OMe	H	H	5.3	3.5
**15 c**	Bn	CO	H	CN	H	H	>100	>100
**16 c**	H	CO	H	CN	H	H	70	37.4
**17 c**	SO_2_NH_2_	CO	H	CN	H	H	25	>100
**15 d**	Bn	CO	H	H	OMe	H	>100	ND^[b]^
**16 d**	H	CO	H	H	OMe	H	>100	ND^[b]^
**17 d**	SO_2_NH_2_	CO	H	H	OMe	H	>100	ND^[b]^
**15 e**	Bn	CO	H	OMe	OMe	H	>100	ND^[b]^
**16 e**	H	CO	H	OMe	OMe	H	2.39	1.6
**17 e**	SO_2_NH_2_	CO	H	OMe	OMe	H	3.4	2.08
**15 f**	Bn	CO	H	OMe	H	OMe	>100	ND^[b]^
**16 f**	H	CO	H	OMe	H	OMe	0.215	0.161
**17 f**	SO_2_NH_2_	CO	H	OMe	H	OMe	0.179	0.118
**15 g**	Bn	CO	H	OMe	OMe	OMe	>100	77.5
**16 g**	H	CO	H	OMe	OMe	OMe	0.051	0.071
**17 g**	SO_2_NH_2_	CO	H	OMe	OMe	OMe	0.166	0.275

[a] Results are the mean of three determinations. [b] Not determined.

Finally, 3′,4′,5′-trimethoxybenzoyl DHIQ derivatives **15 g**, **16 g** and **17 g** were also evaluated. From our understanding drawn from experience with the THIQ systems these compounds could well act as chimeras addressing both the steroidal A-ring binding area and the trimethoxyaryl binding area of the colchicine binding site on tubulin. Phenol **16 g** and its sulfamate **17 g** prove to be the most active compounds evaluated, with **16 g** displaying respective GI_50_ values of 51 and 71 nm against the proliferation of DU-145 and MDA MB-231 cells.

Comparing these results with those for related *N*-benzoylated THIQs (Table 2) it is evident that omitting the carbonyl at C1 is in general detrimental towards in vitro antiproliferative activity. Only polymethoxylated compounds are displayed here. The series of 3′,4′-dimethoxy THIQ compounds shows a different trend to the corresponding DHIQ series, because here benzyl-protected compound **14 e** shows the best, but still relatively modest, activity (GI_50_ 26.7 μm in DU-145) with phenol **18 a** and sulfamate **19 a** being surprisingly completely inactive. The 3′,5′-dimethoxy and 3′,4′,5′-trimethoxy derivatives were also evaluated and show a similar trend as their DHIQ counterparts, with phenols **18 b**–**c** showing low micromolar activity that is slightly better than that of the corresponding sulfamates **19 b**–**c**, while benzyl-protected compounds **14 f**–**g** display only modest activity. However, the most active compounds **18 c** and **19 c** are still about 10- to 20-fold less active than DHIQs **16 g** and **17 g**, showing the importance of the combination of two carbonyl groups (at C1 and in the linker to the D-ring mimic) to achieve compounds with low nanomolar activity.

**Table 2:** Antiproliferative activity of polymethoxylated *N*-benzoyl THIQs against DU-145 human prostate cancer cells and MDA MB-231 human breast cancer cells in vitro

Compd	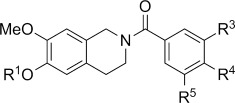				GI_50_ [μm]^[a]^	
	R^1^	R^3^	R^4^	R^5^	DU-145	MDA MB-231
**14 e**	Bn	OMe	OMe	H	26.7	ND^[b]^
**18 a**	H	OMe	OMe	H	>100	ND^[b]^
**19 a**	SO_2_NH_2_	OMe	OMe	H	>100	ND^[b]^
**14 f**	Bn	OMe	H	OMe	46.8	ND^[b]^
**18 b**	H	OMe	H	OMe	6.4	ND^[b]^
**19 b**	SO_2_NH_2_	OMe	H	OMe	9	ND^[b]^
**14 g**	Bn	OMe	OMe	OMe	62.7	18.9
**18 c**^[c]^	H	OMe	OMe	OMe	0.921	0.733
**19 c**^[c]^	SO_2_NH_2_	OMe	OMe	OMe	1.81	1.72

[a] Results are the mean of three determinations. [b] Not determined. [c] Data for **18 c** and **19 c** are taken from the literature.[8]

The most active compound **16 g** was also tested in the NCI 60-cell line assay (Table 3) that allows activity across a wide range of cancer types to be assessed. Data from seven cell lines are presented along with the mean activity across the whole panel (MGM value). Screening was conducted at concentrations ranging from 10 nm upwards, with maximal activity (where a 50 % growth inhibition was obtained in all cell lines at a concentration of 10 nm) being indicated by an MGM value of 10 nm. The data obtained in the assay confirm the very high potency of **16 g** against a broad range of cancer phenotypes.

**Table 3:** GI_50_ and MGM values of compound 16 g obtained for representative cell lines in the NCI-60 screening panel

Cell Line	GI_50_ [μm]^[a]^	Cell Line	GI_50_ [μm]^[a]^
Leukemia CCRF-CEM	0.0171	Melanoma UACC-62	<0.01
Lung HOP-62	0.0295	Ovarian OVCAR-3	<0.01
Colon HCT-116	0.0216	Renal SN12-C	0.056
CNS SF-539	0.0102	*MGM*	*0.033*

[a] Results are the mean of three determinations. The MGM value (in italics) represents the mean concentration that caused 50 % growth inhibition in all 60 cell lines.

**Figure 2:**
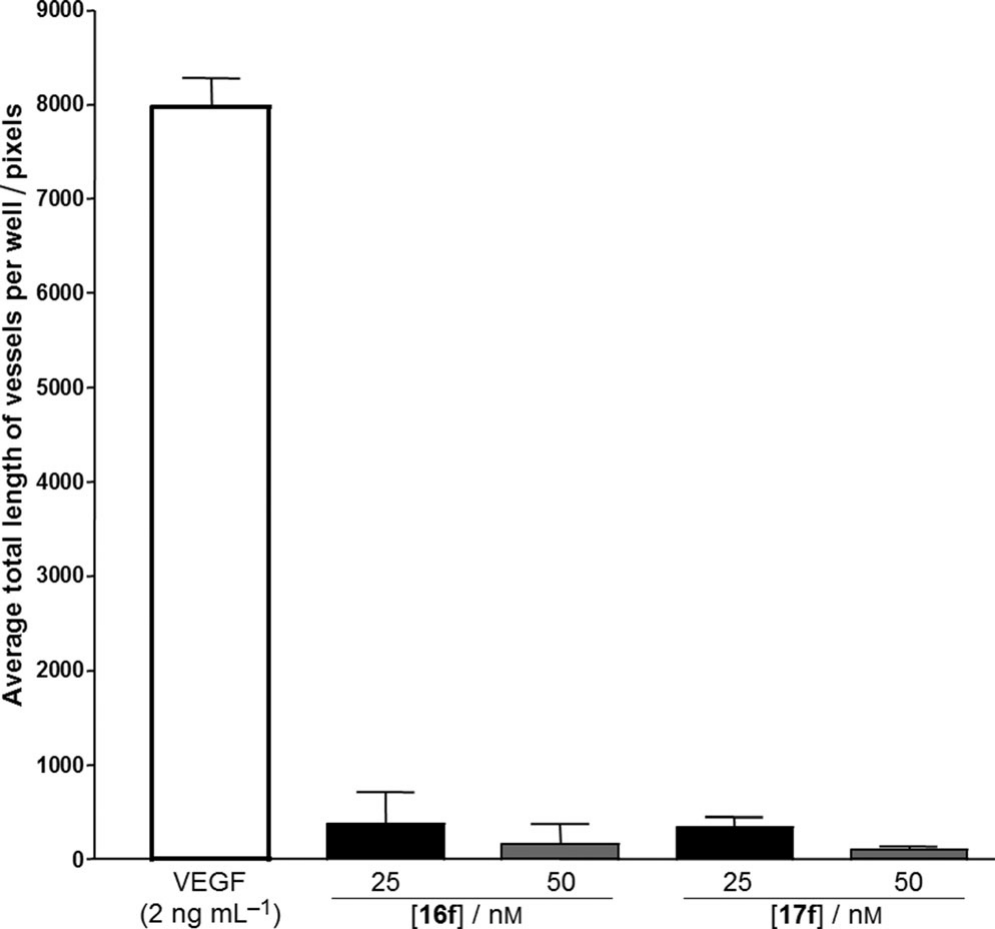
Activity of 16 f and 17 f in an in vitro model of angiogenesis.

As with sulfamates in the tetrahydroisoquinoline series, sulfamate **17 f** also inhibits carbonic anhydrase (IC_50_ 890 nm), which may enhance the bioavailability of this compound.[13] Compounds **16 f** and **17 f** were also assayed for anti-angiogenic activity in an in vitro model of angiogenesis, wherein endothelial cells co-cultured in a matrix of human dermal fibroblasts are used to assess anti-angiogenic potential (Figure 2). When stimulated with VEGF the endothelial cells proliferate and migrate through the matrix to form tubule-like structures. The extent of tubule formation was quantified after 11 days as described elsewhere.[14] Treatment with 25 nm and 50 nm concentrations of **16 f** and **17 f** almost completely inhibits the formation of tubule-like structures (Figure 2). Quantification carried out by calculating total pixel length reflects the lack of tubule formation. Both phenol **16 f** and its sulfamate **17 f** are highly potent.

With these excellent in vitro data in hand, we decided to explore further the microtubule disruptor activity in particular of **16 f**–**g** and **17 f**–**g** alongside the established potent agent combretastatin A-4 (CA-4), currently in clinical trials. All the tested novel compounds show excellent activity as inhibitors of tubulin assembly, with IC_50_ values of 1.2–1.5 μm similar to that of CA-4 (IC_50_ 1.1 μm). Ultimately, as observed before, the concentration required in tubulin-based assays far exceeds the antiproliferative dose, and most likely it suffices to disrupt microtubule dynamics to arrest the cell cycle rather than cause a catastrophic depolymerisation event. It should also be stressed that the nominal compound concentration in antiproliferative assays is that of agent added to the culture medium, rather than the actual concentration within cells, and drug transport can also be a key determinant in reaching the concentration required to achieve an effect. We also determined that **16 f**–**g** and **17 f**–**g** inhibit colchicine binding to tubulin very effectively. Compound **17 f**, which is the most active (89 % inhibition at 5 μm), was also tested at lower concentration and shows 65 % inhibition at 1 μm, approaching again the activity of CA-4 (99 and 90 %). It is thus reasonable to suggest that the activity of the novel DHIQ derivatives can at least partially be ascribed to their ability to disrupt the normal dynamic polymerisation of tubulin by interaction at, or around, the colchicine binding site (Table 4).

**Table 4:** Activity of selected DHIQs as inhibitors of tubulin polymerisation (TP) and colchicine binding (CB) to tubulin

Compd	TP IC_50_ [μm]^[a]^	CB [% inhib.]^[a]^	
		5 μm inhibitor	1 μm inhibitor
CA-4	1.1±0.1	99±0.6	90±0.2
**1 a**^[c]^	2.2±0.3	28±3	ND^[b]^
**1 b**^[c]^	1.3±0.08	78±0.9	ND^[b]^
**1 c**^[c]^	1.3±0.01	45±4	ND^[b]^
**16 f**	1.5±0.1	81±0.7	ND^[b]^
**16 g**	1.3±0.1	83±2	ND^[b]^
**17 f**	1.3±0.01	89±0.1	65±3
**17 g**	1.2±0.03	85±2	ND^[b]^

[a] Values are the mean ±SD of at least two determinations. [b] Not determined. [c] Data for **1 a**–**c** are taken from the literature.[3]

**Figure 3:**
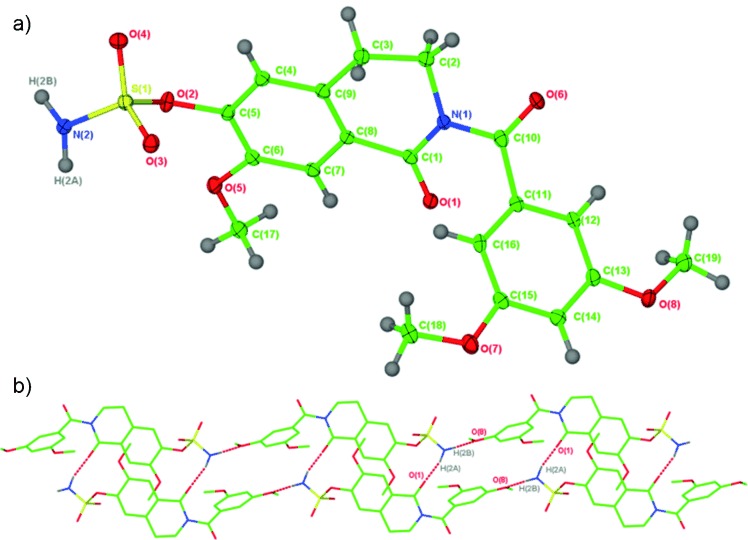
a) Single-crystal X-ray structure of 17 f. Ellipsoids are depicted at 30 % probability. b) Part of the crystal lattice packing diagram of 17 f to illustrate the hydrogen-bonded linear arrangements present in the gross structure.

Finally, an X-ray crystal structure of **17 f** was obtained to explore conformational effects (Figure 3).[15] The crystal structure shows that in the solid state the molecule adopts a “steroid-like” conformation as planned with a dihedral angle of 148.4° between the two carbonyl groups (Figure 3 a). In the single crystal this fixed angle also appears as a result of positive intermolecular interactions (Figure 3 b). The sulfamate group plays a key role to stabilise the framework of the lattice structure by acting as a hydrogen donor to the C3′-methoxy group of a second molecular unit that is part of the same string and to the carbonyl group at C1 of a third molecular unit that is part of a second string running in the opposite direction. The angles in the crystal structure do not necessarily reflect conditions in solution. However, the excellent in vitro antiproliferative activities obtained for this class of compound suggest that adoptable conformations might be very restricted in solution as well, mainly as a result of electrostatic repulsion between the two carbonyl groups, and somewhere around the angle observed in the crystal. Control compounds like **8 d**–**e** and **9 d**–**e** without the carbonyl group in the linker to the D-ring mimic and **18 b**–**c** and **19 b**–**c** without the carbonyl at C1 do not have this favourable restriction and therefore show far less potency than their counterparts **16 f**–**g** and **17 f**–**g** that have both carbonyl groups present.

**Figure 4:**
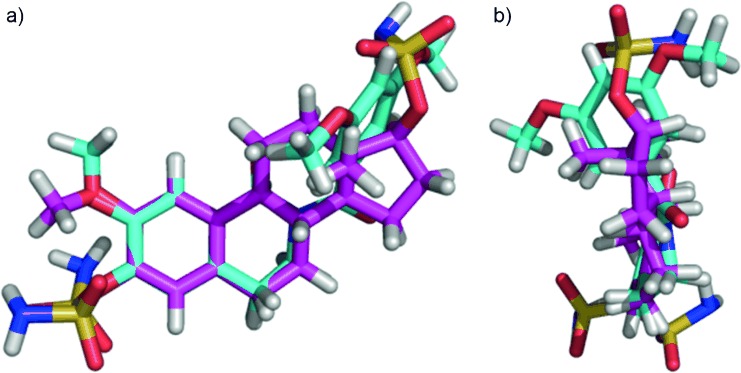
Overlay of the minimum energy conformation of DHIQ 17 f (cyan) with steroid 1 a (pink) viewed from two perspectives.

Molecular structure **17 f** was treated with a computational energy minimisation procedure. These calculations showed, in the minimal energy conformation, that a dihedral angle of 151.0° exists between the two carbonyl groups that is in good agreement with the observed angle of 148.4° in the crystal structure. As we postulated, the 3′,5′-dimethoxybenzoyl group is projected into the area of space that is occupied by the steroidal D ring and the C18 methyl group in the estratriene series. This is illustrated in Figure 4 where the minimum energy state of **17 f** is overlaid with energy-minimised **1 a**. Additionally, the D-ring is rotated 47.2° out of plane of the A,B-ring system leading to one methoxy group being projected into the space of the sulfamate group and the other into the space of the C18 methyl group of **1 a**. Although not shown here, the most potent DHIQs were subjected to the same conformational analysis and the results (angles) obtained were unsurprisingly very similar (**16 g**: 149.8° and 46.9°; **17 g**: 150.5° and 47.1°) to the ones for compound **17 f**. These calculations support our postulate that the presence of the imide sub-structure favours adoption of a “steroid-like” conformation. It seems reasonable to propose that the positive effects on activity can, to some degree, be ascribed to this conformational biasing.

## Conclusions

In summary, dihydroisoquinolinone derivatives including their sulfamate esters are compounds with activities in the low micromolar and nanomolar range against the proliferation of prostate and breast cancer cells. The most potent DHIQ derivative synthesised **16 g**, is also confirmed as having very high potency against a broad range of cancer phenotypes in the NCI 60-cell line assay. As anticipated, these compounds also display anti-angiogenic activity in an in vitro angiogenesis assay and appear to function as microtubule disruptors, as evidenced by their ability to inhibit polymerisation of tubulin and compete at the colchicine binding site. The most active DHIQs **16 f**–**g** and their sulfamates **17 f**–**g** are nearly equipotent to combretastatin A-4 (CA-4) as inhibitors of tubulin assembly and colchicine binding. Additionally, X-ray analysis of **17 f** reveals that electrostatic repulsion between the two adjacent carbonyl groups might partially explain the excellent in vitro activities of this novel series of microtubule disruptors. This conclusion is further supported by molecular modelling studies. Thus, compounds derived from the DHIQ series are worthy of in vivo investigation and potential further pre-clinical development.

## Experimental Section

### In vitro studies

*Cell lines*: DU-145 (brain metastasis carcinoma of the prostate) and MDA MB-231 (metastatic pleural effusion of breast adenocarcinoma) established human cell lines were obtained from ATCC Global Bioresource Center. Cells were maintained in a 5 % CO_2_ humidified atmosphere at 37 °C in RPMI-1640 medium, supplemented with 10 % fetal bovine serum, penicillin, (100 U mL^−1^), and streptomycin (0.1 mg mL^−1^). The effect of drugs on tubule formation was assessed using an angiogenesis kit (TCS-Cellworks).

*Antiproliferative assays*: DU-145 and MDA MB-231 cells were seeded into 96-well microtitre plates (5000 cells per well) and treated with compound (10^−9^–10^−4^ m) or with vehicle control. At 96 h post-treatment, live cell counts were determined by WST-1 cell proliferation assay (Roche, Penzberg, Germany), as per the manufacturer’s instructions. Viability results were expressed as a percentage of mean control values resulting in the calculation of the 50 % growth inhibition (GI_50_). All experiments were performed in triplicate.

*Anti-angiogenic assays*: HUVECs were cultured in a 24-well plate within a matrix of human diploid fibroblasts of dermal origin in optimised medium supplied by the company. The co-cultured cells were incubated throughout the experiment at 37 °C under 5 % CO_2_ in a humidified incubator. On day 1, the culture medium was removed and replaced with medium containing the drug under investigation. On days 4, 7 and 9 the medium was replaced with fresh medium containing the drugs. Suramin (20 μL) was used as a positive anti-angiogenic control and vascular endothelial growth factor (VEGF, 2 ng mL^−1^) as a pro-angiogenic control. Each compound was tested in triplicate. On day 11, the cells were washed (PBS) and 70 % EtOH (1 mL) added to each well for 30 min to fix the cells. After fixation the cells were washed with blocking buffer (1 mL, PBS 1 % BSA; Sigma, UK) and stained for von Willebrand’s factor (manual scoring) for CD31 (computer analysis) in accordance with the manufacturer’s instructions (TCS-Cellworks). The extent of tubule formation was then quantified, either manually by eye or using free software available through the National Institutes of Health website (NIH image [Mac version] from http://rsb.info.nih.gov/nih-image/download.html). For manual scoring a grid was drawn on the back of the plate using a fine marker pen. A 25-point Chalkley eyepiece graticule (Pyser SGI Ltd, UK) was fitted to the microscope and 10x magnification was used to count the tubules within five viewing fields (where the grid lines intersect), the average and SE were then calculated. For computer analysis, eight fixed fields of view for each well (10x magnification) were digitally photographed using a DC120 camera (Kodak, Rochester, NY, USA) with the Photoshop (Adobe, USA) plug-in MDS 120 software (Kodak). The pictures were copied to the NIH image software using Quicktime (Apple, USA) translation. After correcting for background the software counted the number of pixels representing tubules. The total pixel area within the eight fields was then calculated for each well. The average total pixel area and SE were then calculated. There was a significant correlation (*r*=0.94, *p*<0.001) between manual and computer analysis of tubule formation.

*Tubulin assays*: Bovine brain tubulin, prepared as described previously,[16] was used in studies presented here. Assembly IC_50_ values were determined as described in detail elsewhere.[17] Briefly, 1.0 mg mL^−1^ (10 μm) tubulin was pre-incubated without GTP with varying compound concentrations for 15 min at 30 °C. Reaction mixtures were placed on ice, and GTP (final concentration, 0.4 mm) was added. The reaction mixtures were transferred to cuvettes, held at 0 °C in a recording spectrophotometer. Baselines were established at 0 °C, and increase in turbidity was followed for 20 min following a rapid (<30 s) jump to 30 °C. Compound concentrations required to decrease the turbidity increase by 50 % were determined. The method for measuring inhibition of the binding of [^3^H]colchicine to tubulin was described in detail previously.[18] Reaction mixtures contained 0.1 mg mL^−1^ (1.0 μm) tubulin, 5.0 μm [^3^H]colchicine, and potential inhibitor at 5.0 μm. Compounds were compared with CA-4, a particularly potent inhibitor of the binding of colchicine to tubulin.[19] Reaction mixtures were incubated for 10 min at 37 °C, a time point at which the binding of colchicine in control reaction mixtures is generally 40–60 % complete. A minimum of two experiments were performed with each compound.

### Chemistry

**General**: All chemicals were either purchased from Aldrich Chemical Co. (Gillingham, UK) or Alfa Aesar (Heysham, UK). Organic solvents of A.R. grade were supplied by Fisher Scientific (Loughborough, UK) and used as supplied. CHCl_3_, CH_2_Cl_2_, DMA, DMF and THF were purchased from Aldrich and stored under a positive pressure of N_2_ after use. Compounds **13**, **18c** and **19c** were prepared according to literature procedures.[8] Sulfamoyl chloride was prepared by an adaptation of the method of Appel and Berger[20] and was stored in the refrigerator under positive pressure of N_2_ as a solution in toluene as described by Woo et al.[21] An appropriate volume of this solution was freshly concentrated in vacuo immediately before use. Reactions were carried out at room temperature unless stated otherwise. Thin-layer chromatography (TLC) was performed on pre-coated aluminum plates (Merck, silica gel 60 F_254_). Product spots were visualised either by UV irradiation at 254 nm or by staining with either alkaline KMnO_4_ solution or 5 % dodecamolybdophosphoric acid in EtOH, followed by heating. Flash column chromatography was performed using gradient elution (solvents indicated in text) on either pre-packed columns (Isolute) on a Flashmaster II system (Biotage, Uppsala, Sweden) or on a CombiFlash *R*_f_ Automated Flash Chromatography System (Teledyne Isco, Lincoln, NE, USA) with RediSep *R*_f_ disposable flash columns. ^1^H and ^13^C NMR spectra were recorded with either a Delta JMN-GX 270 (Jeol, Peabody, MA, USA) at 270 and 67.5 MHz, respectively, or a Mercury VX 400 NMR spectrometer (Varian, Paolo Alto, CA, USA) at 400 and 100 MHz, respectively. Chemical shifts *δ* are reported in parts per million (ppm) relative to tetramethylsilane (TMS) as internal standard. Coupling constants *J* are recorded to the nearest 0.1 Hz. Mass spectra were recorded at the Mass Spectrometry Service Centre, University of Bath, UK. FAB-MS was carried out using *m*-nitrobenzyl alcohol (NBA) as the matrix. Melting points were determined using a Stuart SMP3 or a Stanford research systems Optimelt MPA100 melting point apparatus (Stanford Research Systems, Sunnyvale, CA, USA) and are uncorrected. All compounds were ≥98 % pure by reversed-phase HPLC run with CH_3_CN/H_2_O or MeOH/H_2_O (Sunfire C_18_ reversed-phase column, 4.6×150 mm, 3.5 μm pore size).

**Crystallographic methods**: Single crystals of compound **17 f** were analysed using a Nonius Kappa CCD diffractometer using Mo(*K*α) radiation. The structure was solved using SHELXS-97[22] and refined using full-matrix least squares in SHELXL-97.[23] The hydrogen atoms attached to N2 were located and refined subject to being a distance of 0.89 Å from the parent atom. CCDC 874341 http://www.ccdc.cam.ac.uk/cgi-bin/catreq.cgicontains the crystallographic data for compound **17 f** and can be obtained free of charge from The Cambridge Crystallographic Data Centre via http://www.ccdc.cam.ac.uk/data_request/cif.

**Computational methods**: The Schrödinger software running under Maestro version 9.2.112 was used for all computational work. The crystal structure of **17 f** solved in this work was used. One molecule was taken from this structure and run through a brief geometry optimisation procedure. Both dihedral angles were manually and independently adjusted in steps of 30° and the energy of the conformers was calculated. Compounds **16 g** and **17 g** were built by altering the crystal structure of **17 f** to yield the desired structure which was then run through a brief geometry optimisation procedure. Conformers were generated and molecular energies calculated as described above.

**5-Hydroxy-6-methoxyindan-1-one (4)**: 5,6-Dimethoxy-indan-1-one (25.93 g, 135.0 mmol) was placed in a 1 L round-bottom flask. Piperidine (200 mL) and H_2_O (50 mL) were added and the reaction mixture was stirred at 140 °C for 8 days. The mixture was concentrated in vacuo and aqueous NaOH (2 m, 400 mL) was added. The mixture was extracted with EtOAc (200 mL) and CH_2_Cl_2_ (200 mL). The aqueous layer was then acidified with HCl (12 m, 100 mL) and extracted with CH_2_Cl_2_ (3×200 mL). This organic layer system was washed with H_2_O (200 mL), dried (MgSO_4_), filtered and concentrated in vacuo to give compound **4** as a yellow powder (16.87 g, 70 %). ^1^H NMR (270 MHz, CDCl_3_): *δ*=2.49–2.60 (2 H, m), 2.86–2.98 (2 H, m), 3.83 (3 H, s), 6.86 (1 H, s), 7.09 (1 H, s), 8.03 ppm (1 H, s, br).

**5-Benzyloxy-6-methoxyindan-1-one (5)**: Compound **4** (3.0 g, 16.8 mmol), benzyl bromide (2.1 mL, 17.7 mmol) and potassium carbonate (4.70 g, 34.0 mmol) were suspended in DMF (20 mL) and stirred at RT for 3 days. H_2_O (50 mL) was added and the mixture was extracted with EtOAc (2×80 mL). The combined organics were washed with H_2_O and brine, dried (MgSO_4_) and concentrated in vacuo. Crystallisation from EtOAc/hexane 2:3 afforded compound **5** as a light-yellow powder (4.10 g, 91 %), mp: 139–140 °C. ^1^H NMR (270 MHz, CDCl_3_): *δ*=2.58–2.66 (2 H, m), 2.93–3.01 (2 H, m), 3.89 (3 H, s), 5.22 (2 H, s), 6.88 (1 H, s), 7.18 (1 H, s), 7.27–7.46 ppm (5 H, m).

**6-Benzyloxy-7-methoxy-3,4-dihydro-2*H*-isoquinolin-1-one (6 a)**: Compound **5** (2.68 g, 10 mmol) was dissolved in CH_2_Cl_2_ (10 mL) and methanesulfonic acid (10 mL) and the solution was cooled to 0 °C. Sodium azide (1.32 g, 20 mmol) was added portionwise over 0.5 h. The mixture was allowed to warm to RT and stirred for 16 h. Aqueous NaOH (1.5 m, 50 mL) was added dropwise at 0 °C. The mixture was extracted with EtOAc (3×80 mL). The combined organics were washed with H_2_O and brine, dried (MgSO_4_) and concentrated in vacuo. Flash column chromatography (hexane/EtOAc 3:1 to EtOAc) and crystallisation from EtOAc afforded compound **6 a** as a white solid (1.45 g, 52 %), mp: 177–178 °C. ^1^H NMR (270 MHz, CDCl_3_): *δ*=2.87 (2 H, t, *J*=7.6 Hz), 3.53 (2 H, t, *J*=7.6 Hz), 3.94 (3 H, s), 5.20 (2 H, s), 6.03 (1 H, s, br), 6.70 (1 H, s), 7.29–7.46 (5 H, m), 7.60 ppm (1 H, s); LC–MS (FAB+): *m*/*z* 284.44 [*M*+H]^+^.

**6-Hydroxy-7-methoxy-3,4-dihydro-2*H*-isoquinolin-1-one (6 b)**: Compound **6 a** (1.418 g, 5.0 mmol) was dissolved in MeOH (50 mL) and filtered. The solution was sucked at 1.0 mL min^−1^ into the H-cube and treated with full hydrogen over Pd/C (10 %, 140 mg cartridge) at RT. The resulting solution was concentrated in vacuo to afford compound **6 b** as a pale-yellow solid (961 mg, 99 %). ^1^H NMR (270 MHz, CDCl_3_): *δ*=2.72 (2 H, t, *J*=6.7 Hz), 3.27–3.31 (2 H, m), 3.77 (3 H, s), 6.65 (1 H, s), 7.33 (1 H, s), 7.64 (1 H, s, br), 9.65 ppm (1 H, s, br); LC–MS (ES+): *m*/*z* 194.2 [*M*+H]^+^.

**7-Methoxy-(6-triisopropyloxy)-3,4-dihydro-2*H*-isoquinolin-1-one (6 c)**: Compound **6 b** (3.1 g, 16 mmol), TIPSCl (7.1 mL, 33 mmol) and imidazole (2.4 g, 35 mmol) in DMF (30 mL) was stirred at RT for 16 h. H_2_O (30 mL) was added and the mixture was extracted with EtOAc (2×80 mL). The combined organic layers were washed with H_2_O and brine, dried (MgSO_4_), filtered and concentrated in vacuo. Flash column chromatography (hexane to EtOAc) afforded compound **6 c** as a white powder (5.0 g, 93 %), mp: 112–113 °C. ^1^H NMR (270 MHz, CDCl_3_): *δ*=1.07 (18 H, d, *J*=6.9 Hz), 1.23 (3 H, sept, *J*=6.9 Hz), 2.85 (2 H, t, *J*=6.7 Hz), 3.51 (2 H, dt, *J*=6.7, 2.9 Hz), 3.82 (3 H, s), 5.86 (1 H, s, br), 6.66 (1 H, s), 7.53 ppm (1 H, s); LC–MS (APCI+): *m*/*z* 350.50 [*M*+H]^+^.

**7-Methoxy-2-(2-methoxybenzyl)-6-(2-methoxybenzyloxy)-3,4-dihydroisoquinolin-1(2*H*)-one** (**7 a)**: Compound **6 c** (505 mg, 1.44 mmol) was dissolved in anhydrous DMF (5 mL) and cooled to 0 °C. Sodium hydride (60 % in mineral oil, 115 mg, 2.88 mmol) was added portionwise and the suspension was stirred at 0 °C for 0.5 h. 2-Methoxybenzyl chloride (0.24 mL, 1.73 mmol) was added dropwise and the reaction mixture was stirred at RT for 4 days. Ammonium chloride (saturated, 10 mL) was added and the mixture was extracted with EtOAc (80 mL). The organic layer was washed with H_2_O and brine, dried (MgSO_4_), filtered and concentrated in vacuo. Flash column chromatography (hexane/EtOAc 10:1 to 2:1) afforded **7 a** as a white powder (350 mg, 56 %), mp: 133–134 °C. ^1^H NMR (270 MHz, CDCl_3_): *δ*=2.81 (2 H, t, *J*=6.7 Hz), 3.50 (2 H, t, *J*=6.7 Hz), 3.83 (3 H, s), 3.85 (3 H, s), 3.93 (3 H, s), 4.78 (2 H, s), 5.22 (2 H, s), 6.64 (1 H, s), 6.84–6.95 (4 H, m), 7.18–7.32 (3 H, m), 7.44 (1 H, dd, *J*=7.4, 1.5 Hz), 7.65 ppm (1 H, s); LC–MS (APCI+): *m*/*z* 434.56 [*M*+H]^+^; HRMS (ES+): *m*/*z* found 434.1960; C_26_H_28_NO_5_^+^ [*M*+H]^+^ requires 434.1962.

**7-Methoxy-2-(3-methoxybenzyl)-6-(triisopropylsilyloxy)-3,4-dihydroisoquinolin-1(2*H*)-one (7b1) and 7-Methoxy-2-(3-methoxybenzyl)-6-(3-methoxybenzyloxy)-3,4-dihydroisoquinolin-1(2*H*)-one (7b2)**: Method as for **7 a** using compound **6 c** (505 mg, 1.44 mmol), sodium hydride (60 %, 115 mg, 2.88 mmol) and 3-methoxybenzyl bromide (0.24 mL, 1.73 mmol) in DMF (5 mL) at 0 °C for 0.5 h and at RT for 4 days. Flash column chromatography (hexane/EtOAc 10:1 to 5:1 to 2:1) afforded **7b1** as a colourless oil (120 mg, 18 %) and **7b2** as a white powder (110 mg, 18 %), mp: 85–86 °C. **7b1**: ^1^H NMR (270 MHz, CDCl_3_): *δ*=1.07 (18 H, d, *J*=6.7 Hz), 1.22 (3 H, sept, *J*=6.7 Hz), 2.79 (2 H, t, *J*=6.7 Hz), 3.44 (2 H, t, *J*=6.7 Hz), 3.77 (3 H, s), 3.84 (3 H, s), 4.73 (2 H, s), 6.61 (1 H, s), 6.80 (1 H, ddd, *J*=8.1, 2.5, 0.8 Hz), 6.85–6.92 (2 H, m), 7.23 (1 H, t, *J*=7.9 Hz), 7.61 ppm (1 H, s); HRMS (ES+): *m*/*z* found 470.2709; C_27_H_40_NO_4_Si^+^ [*M*+H]^+^ requires 470.2721. **7b2**: ^1^H NMR (270 MHz, CDCl_3_): *δ*=2.77 (2 H, t, *J*=6.6 Hz), 3.41 (2 H, t, *J*=6.6 Hz), 3.75 (3 H, s), 3.77 (3 H, s), 3.92 (3 H, s), 4.72 (2 H, s), 5.14 (2 H, s), 6.61 (1 H, s), 6.76–6.89 (4 H, m), 6.94–7.01 (2 H, m), 7.21 (1 H, t, *J*=7.9 Hz), 7.25 (1 H, t, *J*=8.1 Hz), 7.66 ppm (1 H, s); LC–MS (APCI+): *m*/*z* 434.56 [*M*+H]^+^; HRMS (ES+): *m*/*z* found 434.1962; C_26_H_28_NO_5_^+^ [*M*+H]^+^ requires 434.1962.

**7-Methoxy-6-(triisopropylsilyloxy)-2-(4-methoxybenzyl)-3,4-dihydroisoquinolin-1(2*H*)-one (7 c)**: Method as for **7 a** using compound **6 c** (524 mg, 1.5 mmol), sodium hydride (60 %, 64 mg, 1.6 mmol) and 4-methoxybenzyl bromide (0.26 mL, 1.8 mmol) in DMF (5 mL) at 0 °C for 0.5 h and at RT for 6 h. Flash column chromatography (hexane/EtOAc 10:1 to 5:1) afforded compound **7 c** as a colourless oil (385 mg, 55 %). ^1^H NMR (270 MHz, CDCl_3_): *δ*=1.07 (18 H, d, *J*=6.9 Hz), 1.22 (3 H, sept, *J*=6.9 Hz), 2.77 (2 H, t, *J*=6.7 Hz), 3.42 (2 H, t, *J*=6.7 Hz), 3.78 (3 H, s), 3.84 (3 H, s), 4.68 (2 H, s), 6.60 (1 H, s), 6.84 (2 H, dt, *J*=8.6, 2.4 Hz), 7.26 (2 H, dt, *J*=8.5, 2.3 Hz), 7.60 ppm (1 H, s); LC–MS (APCI+): *m*/*z* 470.57 [*M*+H]^+^; HRMS (ES+): *m*/*z* found 470.2706; C_27_H_40_NO_4_Si^+^ [*M*+H]^+^ requires 470.2721.

**6-Benzyloxy-7-methoxy-2-(3,5-dimethoxybenzyl)-3,4-dihydroisoquinolin-1(2*H*)-one (7 d)**: Method as for **7 a** using compound **6 a** (425 mg, 1.5 mmol), sodium hydride (60 %, 120 mg, 3.0 mmol) and 3,5-dimethoxybenzyl bromide (416 mg, 1.8 mmol) in DMF (10 mL) at 0 °C for 0.5 h and at RT for 18 h. Flash column chromatography (hexane/EtOAc 5:1 to 1:1) gave an oil that was stirred in Et_2_O (50 mL) and hexane (20 mL), filtered and dried in vacuo to afford compound **7 d** as a white powder (440 mg, 68 %), mp: 82–83 °C. ^1^H NMR (270 MHz, CDCl_3_): *δ*=2.79 (2 H, t, *J*=6.7 Hz), 3.43 (2 H, t, *J*=6.7 Hz), 3.75 (6 H, s), 3.93 (3 H, s), 4.69 (2 H, s), 5.17 (2 H, s), 6.35 (1 H, t, *J*=2.5 Hz), 6.45 (2 H, d, *J*=2.5 Hz), 6.62 (1 H, s), 7.27–7.45 (5 H, m), 7.66 ppm (1 H, s); LC–MS (ES+): *m*/*z* 456.03 ([*M*+Na]^+^, 100 %), 434.05 [*M*+H]^+^; HRMS (ES+): *m*/*z* found 434.1956; C_26_H_28_NO_5_^+^ [*M*+H]^+^ requires 434.1962.

**6-Benzyloxy-7-methoxy-2-(3,4,5-trimethoxybenzyl)-3,4-dihydroisoquinolin-1(2*H*)-one (7 e)**: Method as for **7 a** using compound **6 a** (425 mg, 1.5 mmol), sodium hydride (60 %, 120 mg, 3.0 mmol) and 3,4,5-trimethoxybenzyl chloride (390 mg, 1.8 mmol) in DMF (10 mL) at 0 °C for 0.5 h and at RT for 18 h. The residue was stirred in Et_2_O, filtered and dried in vacuo to afford compound **7 e** as a white powder (570 mg, 82 %), mp: 136–137 °C. ^1^H NMR (270 MHz, CDCl_3_): *δ*=2.80 (2 H, t, *J*=6.8 Hz), 3.43 (2 H, t, *J*=6.8 Hz), 3.82 (9 H, s), 3.93 (3 H, s), 4.68 (2 H, s), 5.17 (2 H, s), 6.52 (2 H, s), 6.63 (1 H, s), 7.26–7.43 (5 H, m), 7.66 ppm (1 H, s); LC–MS (ES+): *m*/*z* 486.26 ([*M*+Na]^+^, 100 %), 464.28 [*M*+H]^+^; HRMS (ES+): *m*/*z* found 464.2064; C_27_H_30_NO_6_^+^ [*M*+H]^+^ requires 464.2068.

**6-Hydroxy-7-methoxy-2-(2-methoxybenzyl)-3,4-dihydroisoquinolin-1(2*H*)-one (8 a)**: A mixture of compound **7 a** (290 mg, 0.67 mmol) and Pd/C (10 %, 40 mg) in THF (20 mL) and MeOH (20 mL) was stirred under hydrogen at RT for 4 h. After filtration through Celite and evaporation under reduced pressure the residue was stirred in Et_2_O, filtered and dried in vacuo to afford compound **8 a** as a white powder (195 mg, 93 %), mp: 164–165 °C. ^1^H NMR (270 MHz, CDCl_3_): *δ*=2.83 (2 H, t, *J*=6.7 Hz), 3.51 (2 H, t, *J*=6.7 Hz), 3.83 (3 H, s), 3.92 (3 H, s), 4.78 (2 H, s), 6.02 (1 H, s), 6.68 (1 H, s), 6.87 (1 H, d, *J*=7.4 Hz), 6.91 (1 H, dd, *J*=7.4, 1.0 Hz), 7.23 (1 H, dt, *J*=7.4, 1.5 Hz), 7.30 (1 H, dd, *J*=7.9, 1.5 Hz), 7.63 ppm (1 H, s); LC–MS (APCI+): *m*/*z* 314.17 [*M*+H]^+^; HRMS (ES+): *m*/*z* found 314.1384; C_18_H_20_NO_4_^+^ [*M*+H]^+^ requires 314.1387.

**6-Hydroxy-7-methoxy-2-(3-methoxybenzyl)-3,4-dihydroisoquinolin-1(2*H*)-one (8 b)**: Method as for **8 a** using compound **7 b2** (85 mg, 0.196 mmol) and Pd/C (10 %, 20 mg) in THF (10 mL) and MeOH (10 mL) under hydrogen at RT for 2 h. Flash column chromatography (hexane/EtOAc 1:1) afforded compound **8 b** as a white powder (52 mg, 76 %), mp: 181–182 °C. ^1^H NMR (270 MHz, CDCl_3_): *δ*=2.82 (2 H, t, *J*=6.7 Hz), 3.44 (2 H, t, *J*=6.7 Hz), 3.77 (3 H, s), 3.93 (3 H, s), 4.74 (2 H, s), 6.03 (1 H, s), 6.68 (1 H, s), 6.80 (1 H, dd, *J*=7.4, 1.7 Hz), 6.84 (1 H, d, *J*=1.7 Hz), 6.90 (1 H, d, *J*=7.7 Hz), 7.23 (1 H, t, *J*=7.9 Hz), 7.64 ppm (1 H, s); LC–MS (APCI+): *m*/*z* 314.23 [*M*+H]^+^; HRMS (ES+): *m*/*z* found 314.1383, C_18_H_20_NO_4_^+^ [*M*+H]^+^ requires 314.1387.

**6-Hydroxy-7-methoxy-2-(3-methoxybenzyl)-3,4-dihydroisoquinolin-1(2*H*)-one (8 b)**: Compound **7 b1** (110 mg, 0.23 mmol) was dissolved in THF (20 mL). TBAF (1.0 m in THF, 028 mL, 0.28 mmol) was added dropwise and the reaction mixture was stirred at RT for 2 h. H_2_O was added and the mixture extracted with EtOAc. The organic layer was washed with H_2_O, brine, dried (MgSO_4_) and concentrated in vacuo. The resulting yellow solid was stirred in Et_2_O, filtered and dried in vacuo to afford compound **8 b** as a white powder (60 mg, 82 %). Analytical data are identical as shown above.

**6-Hydroxy-7-methoxy-2-(4-methoxybenzyl)-3,4-dihydroisoquinolin-1(2*H*)-one (8 c)**: Method as for **8 b** using compound **7 c** (0.24 g, 0.51 mmol) and TBAF (1.0 m in THF, 0.61 mL, 0.61 mmol) in THF (10 mL) at RT for 18 h. Flash column chromatography (hexane/EtOAc 4:1 to 2:1) afforded compound **8 c** as a white powder (110 mg, 69 %), mp: 171–172 °C. ^1^H NMR (270 MHz, CDCl_3_): *δ*=2.78 (2 H, t, *J*=6.7 Hz), 3.41 (2 H, t, *J*=6.7 Hz), 3.77 (3 H, s), 3.90 (3 H, s), 4.69 (2 H, s), 6.33 (1 H, s, br), 6.66 (1 H, s), 6.84 (2 H, dt, *J*=8.7, 2.3 Hz), 7.24 (2 H, dt, *J*=8.9, 2.3 Hz), 7.63 ppm (1 H, s); LC–MS (APCI+): *m*/*z* 314.42 [*M*+H]^+^; HRMS (ES+): *m*/*z* found 314.1381; C_18_H_19_NO_4_^+^ [*M*+H]^+^ requires 314.1387.

**6-Hydroxy-7-methoxy-2-(3,5-dimethoxybenzyl)-3,4-dihydroisoquinolin-1(2*H*)-one (8 d)**: Method as for **8 a** using compound **7 d** (330 mg, 0.76 mmol) and Pd/C (10 %, 40 mg) in THF (20 mL) and MeOH (30 mL) under hydrogen at RT for 18 h. The resulting white solid was stirred in Et_2_O, filtered and dried in vacuo to afford compound **8 d** as a white powder (240 mg, 92 %), mp: 166–167 °C. ^1^H NMR (270 MHz, CDCl_3_): *δ*=2.82 (2 H, t, *J*=6.7 Hz), 3.44 (2 H, t, *J*=6.7 Hz), 3.75 (6 H, s), 3.93 (3 H, s), 4.70 (2 H, s), 6.04 (1 H, s), 6.35 (1 H, t, *J*=2.5 Hz), 6.46 (2 H, d, *J*=2.5 Hz), 6.68 (1 H, s), 7.63 ppm (1 H, s); LC–MS (ES−): *m*/*z* 342.09 [*M*−H]^−^; HRMS (ES+): *m*/*z* found 344.1477; C_19_H_22_NO_5_^+^ [*M*+H]^+^ requires 344.1492.

**6-Hydroxy-7-methoxy-2-(3,4,5-trimethoxybenzyl)-3,4-dihydroisoquinolin-1(2*H*)-one (8 e)**: Method as for **8 a** using compound **7 e** (375 mg, 0.81 mmol) and Pd/C (10 %, 50 mg) in THF (20 mL) and MeOH (20 mL) under hydrogen at RT for 18 h. The resulting white solid was stirred in Et_2_O, filtered and dried in vacuo to afford compound **8 e** as a white powder (260 mg, 86 %), mp: 168–169 °C. ^1^H NMR (270 MHz, CDCl_3_): *δ*=2.82 (2 H, t, *J*=6.7 Hz), 3.44 (2 H, t, *J*=6.7 Hz), 3.82 (9 H, s), 3.92 (3 H, s), 4.69 (2 H, s), 6.08 (1 H, s, br), 6.53 (2 H, s), 6.69 (1 H, s), 7.63 ppm (1 H, s); LC–MS (ES−): *m*/*z* 372.07 [*M*−H]^−^; HRMS (ES+): *m*/*z* found 374.1590; C_20_H_24_NO_6_^+^ [*M*+H]^+^ requires 374.1598.

**7-Methoxy-2-(2-methoxybenzyl)-6-sulfamoyloxy-3,4-dihydroisoquinolin-1(2*H*)-one (9 a)**: Sulfamoyl chloride (0.6 m in toluene, 1.0 mL, 0.6 mmol) was concentrated in vacuo and dissolved in anhydrous DMA (1.0 mL). Compound **8 a** (94 mg, 0.3 mmol) was added as a solid and the reaction mixture was stirred at RT for 18 h. H_2_O (10 mL) was added and the mixture was extracted with EtOAc (2×50 mL). The combined organic layers were washed with H_2_O, brine, dried (MgSO_4_), filtered and concentrated in vacuo. The residue was stirred in Et_2_O, filtered and dried in vacuo to afford compound **9 a** as a white solid (90 mg, 77 %), mp: 142–143 °C. ^1^H NMR (270 MHz, CDCl_3_): *δ*=2.86 (2 H, t, *J*=6.7 Hz), 3.54 (2 H, t, *J*=6.7 Hz), 3.84 (3 H, s), 3.93 (3 H, s), 4.77 (2 H, s), 5.16 (2 H, s), 6.87 (1 H, d, *J*=8.2), 6.91 (1 H, dt, *J*=7.4, 1.0 Hz), 7.14 (1 H, s), 7.21–7.31 (2 H, m), 7.77 ppm (1 H, s); LC–MS (APCI+): *m*/*z* 393.45 [*M*+H]^+^; HRMS (ES+): *m*/*z* found 393.1118; C_18_H_21_N_2_O_6_S^+^ [*M*+H]^+^ requires 393.1115.

**7-Methoxy-2-(3-methoxybenzyl)-6-sulfamoyloxy-3,4-dihydroisoquinolin-1(2*H*)-one (9 b)**: Method as for **9 a** using compound **8 b** (73 mg, 0.23 mmol) and sulfamoyl chloride (0.46 mmol) in DMA at RT for 18 h. Flash column chromatography (hexane/EtOAc 1:1 to 2:3) afforded compound **9 b** as a white solid (60 mg, 67 %), mp: 139–140 °C. ^1^H NMR (270 MHz, CDCl_3_): *δ*=2.86 (2 H, t, *J*=6.7 Hz), 3.47 (2 H, t, *J*=6.7 Hz), 3.78 (3 H, s), 3.94 (3 H, s), 4.73 (2 H, s), 5.19 (2 H, s), 6.79–6.89 (3 H, m), 7.15 (1 H, s), 7.24 (1 H, dt, *J*=7.6, 1.0 Hz), 7.78 ppm (1 H, s); LC–MS (APCI+): *m*/*z* 393.64 [*M*+H]^+^; HRMS (ES+): *m*/*z* found 393.1118; C_18_H_21_N_2_O_6_S^+^ [*M*+H]^+^ requires 393.1115.

**7-Methoxy-2-(4-methoxybenzyl)-6-sulfamoyloxy-3,4-dihydroisoquinolin-1(2*H*)-one (9 c)**: Method as for **9 a** using compound **8 c** (82 mg, 0.26 mmol) and sulfamoyl chloride (0.78 mmol) in DMA (1.0 mL) at RT for 24 h. Flash column chromatography (hexane/EtOAc 3:1 to 1:1) gave a solid that was stirred in Et_2_O (10 mL), filtered and dried in vacuo to afford compound **9 c** as a white solid (75 mg, 73 %), mp: 159–160 °C. ^1^H NMR (270 MHz, CDCl_3_/[D_4_]MeOH 10:1): *δ*=2.12 (2 H, s), 2.81 (2 H, t, *J*=6.7 Hz), 3.42 (2 H, t, *J*=6.7 Hz), 3.76 (3 H, s), 3.90 (3 H, s), 4.67 (2 H, s), 6.83 (2 H, dt, *J*=8.9, 2.5 Hz), 7.14 (1 H, s), 7.20 (2 H, dt, *J*=8.9, 2.5 Hz), 7.72 ppm (1 H, s); LC–MS (APCI+): *m*/*z* 393.38 [*M*+H]^+^; HRMS (ES+): *m*/*z* found 393.1117; C_18_H_21_N_2_O_6_S^+^ [*M*+H]^+^ requires 393.1115.

**7-Methoxy-2-(3,5-dimethoxybenzyl)-6-sulfamoyloxy-3,4-dihydroisoquinolin-1(2*H*)-one (9 d)**: Method as for **9 a** using compound **8 d** (100 mg, 0.29 mmol) and sulfamoyl chloride (0.87 mmol) in DMA (1.0 mL) at RT for 24 h. The residue was stirred in Et_2_O, filtered, washed with Et_2_O and dried in vacuo to afford compound **9 d** as a white powder (95 mg, 77 %), mp: 164–165 °C. ^1^H NMR (270 MHz, CDCl_3_): *δ*=2.90 (2 H, t, *J*=6.6 Hz), 3.49 (2 H, t, *J*=6.6 Hz), 3.74 (6 H, s), 3.84 (3 H, s), 4.64 (2 H, s), 6.40–6.45 (3 H, s), 7.26 (1 H, s), 7.61 (1 H, s), 8.08 ppm (2 H, s, br); LC–MS (ES−): *m*/*z* 421.13 [*M*−H]^−^; HRMS (ES+): *m*/*z* found 423.1215; C_19_H_23_N_2_O_7_S^+^ [*M*+H]^+^ requires 423.1220.

**7-Methoxy-6-sulfamoyloxy-2-(3,4,5-trimethoxybenzyl)-3,4-dihydroisoquinolin-1(2*H*)-one (9 e)**: Method as for **9 a** using compound **8 e** (100 mg, 0.27 mmol) and sulfamoyl chloride (0.8 mmol) in DMA (1.0 mL) at RT for 24 h. After addition of H_2_O (20 mL), EtOAc (100 mL) and THF (50 mL) the organics could not be dissolved. The solvents were evaporated and the resultant solid in the aqueous layer was washed, filtered, washed with H_2_O, Et_2_O, EtOAc and dried in vacuo to afford compound **9 e** as a white powder (90 mg, 74 %), mp: 201–203 °C. ^1^H NMR (270 MHz, [D_6_]DMSO): *δ*=2.90 (2 H, t, *J*=6.6 Hz), 3.49 (2 H, t, *J*=6.6 Hz), 3.63 (3 H, s), 3.74 (6 H, s), 3.84 (3 H, s), 4.64 (2 H, s), 6.61 (2 H, s), 7.26 (1 H, s), 7.61 (1 H, s), 8.08 ppm (2 H, s, br); LC–MS (ES−): *m*/*z* 451.18 [*M*−H]^−^; HRMS (ES+): *m*/*z* found 453.1313; C_20_H_25_N_2_O_8_S^+^ [*M*+H]^+^ requires 453.1326.

**6-Benzyloxy-7-methoxy-2-((2-methoxyphenyl)sulfonyl)-3,4-dihydroisoquinolin-1(2*H*)-one (10 a)**: Sodium hydride (60 % in mineral oil, 46 mg, 1.9 mmol) was suspended in anhydrous DMF (5 mL). Compound **6 a** (300 mg, 1.0 mmol) was added and the reaction mixture was heated at 50 °C for 0.5 h. The reaction mixture was cooled to RT and 2-methoxybenzenesulfonyl chloride (0.15 mL, 1.0 mmol) was added dropwise. The reaction mixture was stirred for 3.5 h and turned from yellow to almost colourless after addition of the sulfonyl chloride. A further 0.5 equiv (0.07 mL) of the sulfonyl chloride was added and the reaction mixture stirred for a further 2 h. The reaction mixture was poured into sodium bicarbonate (sat., 100 mL) and extracted with CHCl_3_ (3×50 mL). The combined organic layers were washed with H_2_O (4×50 mL) and brine (50 mL), dried (MgSO_4_) and concentrated in vacuo. Flash column chromatography (hexane/EtOAc 2:1) afforded the compound **10 a** as a colourless foam (314 mg, 65 %), mp: 202–203 °C. ^1^H NMR (270 MHz, CDCl_3_): *δ*=2.99 (2 H, t, *J*=6.3 Hz), 3.80 (3 H, s), 3.88 (3 H, s), 4.24 (2 H, t, *J*=6.3 Hz), 5.19 (2 H, s), 6.66 (1 H, s), 6.96 (1 H, d, *J*=8.2 Hz), 7.14 (1 H, dt, *J*=7.6, 1.0 Hz), 7.31–7.44 (6 H, m), 7.52–7.58 (1 H, m), 8.20 ppm (1 H, dd, *J*=7.9, 1.7 Hz); LC–MS (ES+): *m*/*z* 454.46 [*M*+H]^+^; HRMS (ES+): *m*/*z* found 476.1124; C_24_H_23_NO_6_SNa^+^ [*M*+Na]^+^ requires 476.1144.

**6-Benzyloxy-7-methoxy-2-((3-methoxyphenyl)sulfonyl)-3,4-dihydroisoquinolin-1(2*H*)-one (10 b)**: Method as for **10 a** using compound **6 a** (300 mg, 1.1 mmol), sodium hydride (60 % in mineral oil, 64 mg, 1.9 mmol) and 3-methoxybenzenesulfonyl chloride (0.22 mL, 1.6 mmol) in anhydrous DMF (5 mL) at 50 °C for 0.5 h and at RT for 2 h and at 40 °C for 2 h. Flash column chromatography (hexane/EtOAc 2:1) afforded compound **10 b** as a colourless foam (369 mg, 77 %), mp: 140–145 °C. ^1^H NMR (270 MHz, CDCl_3_): *δ*=2.99 (2 H, t, *J*=6.2 Hz), 3.84 (3 H, s), 3.87 (3 H, s), 4.18 (2 H, t, *J*=6.2 Hz), 5.18 (2 H, s), 6.64 (1 H, s), 7.12 (1 H, ddd, *J*=8.2, 2.7, 1.0 Hz), 7.31–7.44 (6 H, m), 7.47 (1 H, s), 7.58–7.63 ppm (2 H, m); LC–MS (ES+): *m*/*z* 476.50 [*M*+Na]^+^; HRMS (ES+): *m*/*z* found 476.1116; C_24_H_23_NO_6_SNa^+^ [*M*+Na]^+^ requires 476.1144.

**6-Benzyloxy-7-methoxy-2-((4-methoxyphenyl)sulfonyl)-3,4-dihydroisoquinolin-1(2*H*)-one (10 c)**: Method as for **10 a** using compound **6 a** (300 mg, 1.0 mmol), sodium hydride (60 % in mineral oil, 46 mg, 1.9 mmol) and 3-methoxybenzenesulfonyl chloride (0.22 mL, 1.5 mmol) in anhydrous DMF (5 mL) at 50 °C for 0.5 h and at RT for 6 h. Flash column chromatography (hexane/EtOAc 2:1) afforded compound **10 c** as a colourless oil (152 mg, 32 %). ^1^H NMR (270 MHz, CDCl_3_): *δ*=2.97 (2 H, t, *J*=6.3 Hz), 3.82 (3 H, s), 3.87 (3 H, s), 4.16 (2 H, t, *J*=6.3 Hz), 5.17 (2 H, s), 6.63 (1 H, s), 6.95–6.99 (2 H, m), 7.28–7.41 (5 H, m), 7.46 (1 H, s), 7.98–8.03 ppm (2 H, m); LC–MS (ES+): *m*/*z* 454.60 [*M*+H]^+^; HRMS (ES+): *m*/*z* found 454.1320; C_24_H_24_NO_6_S^+^ [*M*+H]^+^ requires 454.1324.

**6-Benzyloxy-7-methoxy-2-((3-chlorophenyl)sulfonyl)-3,4-dihydroisoquinolin-1(2*H*)-one (10 d)**: Method as for **10 a** using compound **6 a** (300 mg, 1.0 mmol), sodium hydride (60 % in mineral oil, 46 mg, 1.9 mmol) and 3-methoxybenzenesulfonyl chloride (0.22 mL, 1.5 mmol) in anhydrous DMF (5 mL) at 50 °C for 0.5 h and at RT for 6 h. Flash column chromatography (hexane/EtOAc 2:1) afforded compound **10 d** as a yellow foam (259 mg, 54 %), mp: 155–158 °C. ^1^H NMR (270 MHz, CDCl_3_): *δ*=3.00 (2 H, t, *J*=6.2 Hz), 3.83 (3 H, s), 4.18 (2 H, t, *J*=6.2 Hz), 5.18 (2 H, s), 6.65 (1 H, s), 7.27–7.49 (7 H, m), 7.57 (1 H, ddd, *J*=7.9, 2.0, 1.2 Hz), 7.96–8.01 ppm (2 H, m); LC–MS (ES+): *m*/*z* 458.52 [*M*+H]^+^; HRMS (ES+): *m*/*z* found 480.0621; C_23_H_20_ClNO_5_SNa^+^ [*M*+Na]^+^ requires 480.0643.

**Methyl 2-((6-benzyloxy-7-methoxy-1-oxo-3,4-dihydroisoquinolin-2(1*H*)-yl)sulfonyl)benzoate (10 e)**: Method as for **10 a** using compound **6 a** (300 mg, 1.0 mmol), sodium hydride (60 % in mineral oil, 46 mg, 1.9 mmol) and 3-methoxybenzenesulfonyl chloride (0.22 mL, 1.5 mmol) in anhydrous DMF (5 mL) at 50 °C for 0.5 h and at RT for 6 h. Purification by flash column chromatography (hexane/EtOAc 2:1) afforded compound **10 e** as a colourless powder (176 mg, 35 %). ^1^H NMR (270 MHz, CDCl_3_): *δ*=3.05 (2 H, t, *J*=6.2 Hz), 3.83 (3 H, s), 3.92 (3 H, s), 4.19 (2 H, t, *J*=6.2 Hz), 5.18 (2 H, s), 6.65 (1 H, s), 7.28–7.41 (5 H, m), 7.46 (1 H, s), 7.61–7.71 (3 H, m), 8.55 ppm (1 H, dd, *J*=6.4, 2.0 Hz); LC–MS (APCI-): *m*/*z* 482.29 [*M*−H]^−^; HRMS (ES+): *m*/*z* found 504.1075; C_25_H_23_NO_7_SNa^+^ [*M*+Na]^+^ requires 504.1087.

**6-Hydroxy-7-methoxy-2-((2-methoxyphenyl)sulfonyl)-3,4-dihydroisoquinolin-1(2*H*)-one (11 a)**: Method as for **8 a** using compound **10 a** (316 mg, 0.7 mmol) and Pd/C (10 %, 32 mg) in THF (3 mL) and EtOH (3 mL) under hydrogen at RT for 2 h. The residue was crystallised from dichloromethane/hexane to afford compound **11 a** as a colourless powder (145 mg, 57 %), mp: 222–224 °C. ^1^H NMR (270 MHz, CDCl_3_): *δ*=3.02 (2 H, t, *J*=6.3 Hz), 3.81 (3 H, s), 3.88 (3 H, s), 4.25 (2 H, t, *J*=6.3 Hz), 6.07 (1 H, s), 6.72 (1 H, s), 6.96 (1 H, d, *J*=8.4 Hz), 7.12 (1 H, t, *J*=7.7 Hz), 7.42 (1 H, s), 7.51–7.58 (1 H, m), 8.19 ppm (1 H, dd, *J*=7.9, 1.7 Hz); LC–MS (ES−): *m*/*z* 362.33 [*M*−H]^−^; HRMS (ES+): *m*/*z* found 386.0651; C_17_H_17_NO_6_SNa^+^ [*M*+Na]^+^ requires 386.0674.

**6-Hydroxy-7-methoxy-2-((3-methoxyphenyl)sulfonyl)-3,4-dihydroisoquinolin-1(2*H*)-one (11 b)**: **M**ethod as for **8 a** using compound **10 b** (369 mg, 0.81 mmol) and Pd/C (10 %, 37 mg) in THF (5 mL) and EtOH (5 mL) under hydrogen at RT for 2 h. Flash column chromatography (hexane/EtOAc 1:1 to 1:2 to 1:3) afforded compound **11 b** as a colourless solid (203 mg, 69 %), mp: 195–197 °C. ^1^H NMR (270 MHz, CDCl_3_): *δ*=3.02 (2 H, t, *J*=6.3 Hz), 3.84 (3 H, s), 3.86 (3 H, s), 4.19 (2 H, t, *J*=6.3 Hz), 6.07 (1 H, s), 6.71 (1 H, s), 7.11 (1 H, ddd, *J*=8.4, 2.6, 1.0 Hz), 7.41 (1 H, t, *J*=8.1 Hz), 7.45 (1 H, s), 7.57–7.63 ppm (2 H, m); LC–MS (ES−): *m*/*z* 362.42 [*M*−H]^−^; HRMS (ES+): *m*/*z* found 386.0657; C_17_H_17_NO_6_SNa^+^ [*M*+Na]^+^ requires 386.0674.

**6-Hydroxy-7-methoxy-2-((4-methoxyphenyl)sulfonyl)-3,4-dihydroisoquinolin-1(2*H*)-one (11 c)**: Method as for **8 a** using compound **10 c** (150 mg, 0.33 mmol) and Pd/C (10 %, 15 mg) in THF (5 mL) and EtOH (5 mL) under hydrogen at RT for 3 h. Flash column chromatography (hexane/EtOAc 2:1) afforded compound **11 c** as a colourless powder (74 mg, 62 %), mp: 202–204 °C. ^1^H NMR (270 MHz, CDCl_3_): *δ*=3.00 (2 H, t, *J*=6.3 Hz), 3.84 (3 H, s), 3.85 (3 H, s), 4.17 (2 H, t, *J*=6.2 Hz), 6.08 (1 H, s), 6.70 (1 H, s), 6.95–6.99 (2 H, m), 7.45 (1 H, s), 7.99–8.03 ppm (2 H, m); LC–MS (ES−): *m*/*z* 362.33 [*M*−H]^−^; HRMS (ES+): *m*/*z* found 386.0651; C_17_H_17_NO_6_SNa^+^ [*M*+Na]^+^ requires 386.0674.

**6-Hydroxy-7-methoxy-2-((3-chlorophenyl)sulfonyl)-3,4-dihydroisoquinolin-1(2*H*)-one (11 d)**: Method as for **8 a** using compound **10 d** (240 mg, 0.53 mmol) and Pd/C (10 %, 24 mg) in THF (5 mL) and EtOH (5 mL) under hydrogen at RT for 1 h. Flash column chromatography (hexane to EtOAc) afforded compound **11 d** as a colourless solid (158 mg, 82 %), mp: 178–181 °C. ^1^H NMR (270 MHz, CDCl_3_): *δ*=3.03 (2 H, t, *J*=6.2 Hz), 3.85 (3 H, s), 4.19 (2 H, t, *J*=6.3), 6.11 (1 H, s), 6.72 (1 H, s), 7.44 (1 H, s), 7.44–7.50 (1 H, m), 7.54–7.59 (1 H, m), 7.96–8.00 (1 H, m), 8.02 ppm (1 H, t, *J*=1.5 Hz); LC–MS (ES−): *m*/*z* 366.46 [*M*−H]^−^; HRMS (ES+): *m*/*z* found 390.0160; C_16_H_14_ClNO_5_SNa^+^ [*M*+Na]^+^ requires 390.0173.

**Methyl 2-((6-hydroxy-7-methoxy-1-oxo-3,4-dihydroisoquinolin-2(1*H*)-yl)sulfonyl)benzoate (11 e)**: Method as for **8 a** using compound **10 e** (160 mg, 0.33 mmol) and Pd/C (10 %, 90 mg) in THF (5 mL) and EtOH (5 mL) under hydrogen at RT for 3 h. Flash column chromatography (hexane to EtOAc) afforded compound **11 e** as a colourless solid (99 mg, 76 %), mp: 236–239 °C. ^1^H NMR (270 MHz, CDCl_3_): *δ*=3.08 (2 H, t, *J*=6.2 Hz), 3.83 (3 H, s), 3.93 (3 H, s), 4.19 (2 H, t, *J*=6.2 Hz), 6.08 (1 H, s), 6.71 (1 H, s), 7.44 (1 H, s), 7.60–7.70 (3 H, m), 8.54 ppm (1 H, dd, *J*=6.2, 1.7 Hz); LC–MS (APCI-): *m*/*z* 390.08 [*M*−H]^−^; HRMS (ES+): *m*/*z* found 414.0609; C_18_H_17_NO_7_SNa^+^ [*M*+Na]^+^ requires 414.0618.

**7-Methoxy-2-((2-methoxyphenyl)sulfonyl)-6-(sulfamoyloxy)-3,4-dihydroisoquinolin-1(2*H*)-one (12 a)**: Method as for **9 a** using compound **11 a** (126 mg, 0.35 mmol) and sulfamoyl chloride (0.69 mmol) in anhydrous DMA (1.0 mL) at RT for 22 h. Flash column chromatography (hexane to EtOAc) afforded compound **12 a** as a white powder (115 mg, 75 %), mp: 195–197 °C. ^1^H NMR (270 MHz, [D_6_]DMSO): *δ*=3.09 (2 H, t, *J*=6.2 Hz), 3.78 (3 H, s), 3.89 (3 H, s), 4.18 (2 H, t, *J*=6.2 Hz), 7.18 (1 H, t, *J*=7.7 Hz), 7.24 (1 H, d, *J*=8.4 Hz), 7.37 (1 H, s), 7.44 (1 H, s), 7.68 (1 H, td, *J*=8.4, 1.6 Hz), 7.79 (1 H, dd, *J*=7.8, 1.6 Hz), 8.14 ppm (2 H, s, br); LC–MS (ES−): *m*/*z* 441.17 [*M*−H]^−^; HRMS (ES+): *m*/*z* found 465.0394; C_17_H_18_N_2_O_8_S_2_Na^+^ [*M*+Na]^+^ requires 465.0402.

**7-Methoxy-2-((3-methoxyphenyl)sulfonyl)-6-(sulfamoyloxy)-3,4-dihydroisoquinolin-1(2*H*)-one (12 b)**: Method as for **9 a** using compound **11 b** (200 mg, 0.55 mmol) and sulfamoyl chloride (2.2 mmol) in anhydrous DMA (3.0 mL) at RT for 24 h. Flash column chromatography (hexane to EtOAc) afforded compound **12 b** as a colourless powder (118 mg, 49 %), mp: 164–166 °C. ^1^H NMR (270 MHz, [D_6_]DMSO): *δ*=3.12 (2 H, t, *J*=6.1 Hz), 3.78 (3 H, s), 3.84 (3 H, s), 4.21 (2 H, t, *J*=6.1 Hz), 7.30 (1 H, dt, *J*=7.6, 2.1 Hz), 7.36 (1 H, s), 7.47 (2 H, s, br), 7.52–7.61 (2 H, m), 8.15 ppm (2 H, s, br); LC–MS (ES−): *m*/*z* 441.38 [*M*−H]^−^; HRMS (ES+): *m*/*z* found 465.0382; C_17_H_18_N_2_O_8_S_2_Na^+^ [*M*+Na]^+^ requires 465.0402.

**7-Methoxy-2-((4-methoxyphenyl)sulfonyl)-6-(sulfamoyloxy)-3,4-dihydroisoquinolin-1(2*H*)-one (12 c)**: Method as for **9 a** using compound **11 c** (45 mg, 0.13 mmol) and sulfamoyl chloride (0.5 mmol) in anhydrous DMA (1.0 mL) at RT for 18 h. Flash column chromatography (hexane to EtOAc) gave a colourless solid which was stirred in hexane/CH_2_Cl_2_ to afford compound **12 c** as a colourless solid (30 mg, 56 %), mp: 177–180 °C. ^1^H NMR (400 MHz, [D_6_]DMSO): *δ*=3.09 (2 H, t, *J*=6.0 Hz), 3.79 (3 H, s), 3.86 (3 H, s), 4.17 (2 H, t, *J*=6.2 Hz), 7.12–7.16 (2 H, m), 7.34 (1 H, s), 7.46 (1 H, s), 7.94–7.97 (2 H, m), 8.13 ppm (2 H, s, br); LC–MS (ES−): *m*/*z* 441.31 [*M*−H]^−^; HRMS (ES+): *m*/*z* found 465.0392; C_17_H_18_N_2_O_8_S_2_Na^+^ [*M*+Na]^+^ requires 465.0402.

**2-((3-Chlorophenyl)sulfonyl)-7-methoxy-6-(sulfamoyloxy)-3,4-dihydroisoquinolin-1(2*H*)-one (12 d)**: Method as for **9 a** using compound **11 d** (121 mg, 0.33 mmol) and sulfamoyl chloride (0.66 mmol) in anhydrous DMA (1.0 mL) at RT for 22 h. Flash column chromatography (hexane to EtOAc) afforded compound **12 d** as a colourless solid (132 mg, 90 %), mp: 170–173 °C. ^1^H NMR (270 MHz, [D_6_]DMSO): *δ*=3.14 (2 H, t, *J*=6.2 Hz), 3.79 (3 H, s), 4.24 (2 H, t, *J*=6.2 Hz), 7.37 (1 H, s), 7.48 (1 H, s), 7.68 (1 H, t, *J*=8.0 Hz), 7.82–7.85 (1 H, m), 8.00 (1 H, d, *J*=8.2 Hz), 8.06 (1 H, t, *J*=1.8 Hz), 8.17 ppm (2 H, s); LC–MS (ES−): *m*/*z* 445.30 [*M*−H]^−^; HRMS (ES+): *m*/*z* found 468.9894; C_16_H_15_ClN_2_O_7_S_2_Na^+^ [*M*+Na]^+^ requires 468.9901.

**Methyl 2-((7-methoxy-1-oxo-6-(sulfamoyloxy)-3,4-dihydroisoquinolin-2(1*H*)-yl)sulfonyl)benzoate (12 e)**: Method as for **9 a** using compound **11 e** (68 mg, 0.17 mmol) and sulfamoyl chloride (0.52 mmol) in anhydrous DMA (1.0 mL) at RT for 20 h. Flash column chromatography (hexane to EtOAc) afforded compound **12 e** as a colourless powder (49 mg, 60 %), mp: 181–186 °C. ^1^H NMR (270 MHz, [D_6_]DMSO): *δ*=3.14 (2 H, t, *J*=5.8 Hz), 3.80 (3 H, s), 3.88 (3 H, s), 4.13 (2 H, t, *J*=5.8 Hz), 7.37 (1 H, s), 7.49 (1 H, s), 7.72–7.87 (3 H, m), 8.16 (2 H, s, br), 8.32–8.35 ppm (1 H, m); LC–MS (APCI-): *m*/*z* 390.02 [*M*−SO_2_NH_2_]^−^; HRMS (ES+): *m*/*z* found 493.0343; C_18_H_18_N_2_O_9_S_2_Na^+^ [*M*+Na]^+^ requires 493.0346.

**6-Benzyloxy-2-(2-methoxybenzoyl)-7-methoxy-1,2,3,4-tetrahydroisoquinoline (14 a)**: Compound **13** (325 mg, 1.2 mmol) was dissolved in CHCl_3_ (20 mL) and Et_3_N (1.0 mL, 7.2 mmol). 2-Methoxybenzoyl chloride (239 mg, 1.4 mmol) was added portionwise. The reaction mixture was stirred at RT for 16 h and washed with H_2_O and brine, dried (MgSO_4_), filtered and concentrated in vacuo. Flash column chromatography (hexane/EtOAc 3:1 to 2:3) afforded compound **14 a** as a white powder (405 mg, 84 %), mp: 92–93 °C. ^1^H NMR (270 MHz, CDCl_3_): *δ*=2.58–2.70 and 2.76–2.86 (2 H, m), 3.38–3.48 and 3.74–3.94 (2 H, m), 3.72, 3.76, 3.81 and 3.88 (6 H, s), 4.21–4.42 and 4.76–4.93 (2 H, m), 5.11 (2 H, s), 6.38 and 6.61 (1 H, s), 6.67 and 6.69 (1 H, s), 6.92 (1 H, d, *J*=8.2 Hz), 6.99 (1 H, t, *J*=7.4 Hz), 7.22–7.46 ppm (7 H, m); LC–MS (APCI+): *m*/*z* 402.51 (M^+^-H), *m*/*z* 404.46 [*M*+H]^+^; HRMS (ES+): *m*/*z* found 404.1856; C_25_H_26_NO_4_^+^ [*M*+H]^+^ requires 404.1856.

**6-Benzyloxy-2-(3-methoxybenzoyl)-7-methoxy-1,2,3,4-tetrahydroisoquinoline (14 b)**: Method as for **14 a** using compound **13** (404 mg, 1.5 mmol), 3-methoxybenzoyl chloride (0.22 mL, 1.65 mmol) and Et_3_N (0.42 mL, 3.0 mmol) in CHCl_3_ (10 mL) at RT for 18 h. The reaction mixture was diluted with EtOAc (80 mL) and washed with H_2_O and brine, dried (MgSO_4_), filtered and concentrated in vacuo. Flash column chromatography (hexane/EtOAc 10:1 to 1:1) afforded compound **14 b** as a thick colourless oil (500 mg, 83 %). ^1^H NMR (270 MHz, CDCl_3_): *δ*=2.66–2.76 and 2.76–2.88 (2 H, m), 3.52–3.64 and 3.72–3.98 (2 H, m), 3.80 (6 H, s), 4.49 and 4.79 (2 H, s), 5.11 (2 H, s), 6.40 and 6.69 (1 H, br), 6.64 (1 H, br), 6.92–7.05 (2 H, m), 6.97 (1 H, s), 7.26–7.46 ppm (6 H, m); LC–MS (APCI+): *m*/*z* 402.45 (M^+^-H), *m*/*z* 404.46 [*M*+H]^+^; HRMS (ES+): *m*/*z* found 404.1858; C_25_H_26_NO_4_^+^ [*M*+H]^+^ requires 404.1856.

**6-Benzyloxy-2-(3-cyanobenzoyl)-7-methoxy-1,2,3,4-tetrahydroisoquinoline (14 c)**: Method as for **14 a** using compound **13** (300 mg, 1.1 mmol), 3-cyanobenzoyl chloride (202 mg, 1.22 mmol) and Et_3_N (1.0 mL, 7.2 mmol) in CHCl_3_ (20 mL) at RT for 2 h. Flash column chromatography (hexane/EtOAc 2:1 to 1:2) afforded compound **14 c** as a white powder (325 mg, 74 %), mp: 156–157 °C. ^1^H NMR (270 MHz, CDCl_3_): *δ*=2.70–2.87 (2 H, br), 3.52–3.57 and 3.91–3.95 (2 H, br), 3.79 and 3.97 (3 H, s), 4.46 and 4.80 (2 H, br), 5.12 (2 H, s), 6.40, 6.65 and 6.68 (2 H, br), 7.27–7.45 (5 H, m), 7.52–7.58 (1 H, m), 7.66–7.75 ppm (3 H, m); LC–MS (ES+): *m*/*z* 421.54 [*M*+Na]^+^.

**6-Benzyloxy-2-(4-methoxybenzoyl)-7-methoxy-1,2,3,4-tetrahydroisoquinoline (14 d)**: Method as for **14 a** using compound **13** (406 mg, 1.5 mmol), 4-methoxybenzoyl chloride (318 mg, 1.8 mmol) and Et_3_N (0.42 mL, 3.0 mmol) in CHCl_3_ (30 mL) at RT for 18 h. The reaction mixture was diluted with EtOAc (80 mL) and washed with H_2_O and brine, dried (MgSO_4_), filtered and concentrated in vacuo. Flash column chromatography (hexane/EtOAc 10:1 to 1:1) afforded compound **14 d** as a white powder (520 mg, 85 %), mp: 126–127 °C. ^1^H NMR (270 MHz, CDCl_3_): *δ*=2.77 (2 H, br), 3.67 (2 H, br), 3.83 (6 H, s), 4.73 (2 H, br), 5.11 (2 H, s), 6.46 and 6.69 (1 H, br), 6.65 (1 H, s), 6.92 (2 H, dt, *J*=8.6, 2.3 Hz), 7.26–7.45 ppm (7 H, m); LC–MS (APCI+): *m*/*z* 404.53 [*M*+H]^+^; HRMS (ES+): *m*/*z* found 404.1858; C_25_H_26_NO_4_^+^ [*M*+H]^+^ requires 404.1856.

**6-Benzyloxy-2-(3,4-dimethoxybenzoyl)-7-methoxy-1,2,3,4-tetra-hydroisoquinoline (14 e)**: Method as for **14 a** using compound **13** (300 mg, 1.1 mmol), 3,4-dimethoxybenzoyl chloride (240 mg, 1.2 mmol) and Et_3_N (0.5 mL, 3.6 mmol) in CHCl_3_ (20 mL) at RT for 18 h. Flash column chromatography (hexane/EtOAc 2:1 to 1:3) afforded compound **14 e** as a white powder (370 mg, 77 %), mp: 126–127 °C. ^1^H NMR (270 MHz, CDCl_3_): *δ*=2.77 (2 H, br), 3.83 (2 H, br), 3.88 (3 H, s), 3.89 (3 H, s), 3.90 (3 H, s), 4.61 and 4.74 (2 H, br), 5.11 (2 H, s), 6.65 (1 H, s), 6.86 (1 H, d, *J*=8.9 Hz), 7.01 (1 H, s), 7.03 (1 H, dd, *J*=8.9, 2.0 Hz), 7.23–7.46 ppm (6 H, m); LC–MS (APCI+): *m*/*z* 432.48 (M^+^-H), *m*/*z* 434.50 [*M*+H]^+^; HRMS (ES+): *m*/*z* found 434.1966; C_26_H_28_NO_5_^+^ [*M*+H]^+^ requires 434.1962.

**6-Benzyloxy-2-(3,5-dimethoxybenzoyl)-7-methoxy-1,2,3,4-tetra-hydroisoquinoline (14 f)**: Method as for **14 a** using compound **13** (404 mg, 1.5 mmol), 3,5-dimethoxybenzoyl chloride (331 mg, 1.65 mmol) and Et_3_N (0.42 mL, 3.0 mmol) in CHCl_3_ (20 mL) at RT for 18 h. The reaction mixture was diluted with EtOAc (80 mL) and washed with H_2_O and brine, dried (MgSO_4_), filtered and concentrated in vacuo. Flash column chromatography (hexane/EtOAc 10:1 to 1:1) afforded compound **14 f** as a white powder (520 mg, 85 %), mp: 171–172 °C. ^1^H NMR (270 MHz, CDCl_3_): *δ*=2.70 and 2.81 (2 H, br), 3.58 and 3.92 (2 H, br), 3.83 (9 H, s), 4.49 and 4.78 (2 H, br), 5.11 (2 H, s), 6.42, 6.63, 6.66 and 6.68 (2 H, br), 6.50 (1 H, t, *J*=2.2 Hz), 6.55 (2 H, d, *J*=2.2 Hz), 7.26–7.45 ppm (5 H, m); LC–MS (APCI+): *m*/*z* 434.43 [*M*+H]^+^; HRMS (ES+): *m*/*z* found 434.1962; C_26_H_28_NO_5_^+^ [*M*+H]^+^ requires 434.1962.

**6-Benzyloxy-7-methoxy-2-(3,4,5-trimethoxybenzoyl)-1,2,3,4-tetrahydroisoquinoline (14 g)**: Method as for **14 a** using compound **13** (808 mg, 3.0 mmol), 3,4,5-trimethoxybenzoyl chloride (765 mg, 3.3 mmol) and Et_3_N (2.0 mL, 14.4 mmol) in CHCl_3_ (20 mL) at RT for 18 h. The reaction mixture was diluted with CHCl_3_ (80 mL) and washed with H_2_O and brine, dried (MgSO_4_), filtered and concentrated in vacuo. Flash column chromatography (hexane/EtOAc 3:1 to 1:2) afforded compound **14 g** as a white powder (1.10 g, 79 %), mp: 63–64 °C. ^1^H NMR (270 MHz, CDCl_3_): *δ*=2.80 (2 H, br), 3.60 and 3.80 (2 H, br), 3.85 (6 H, s), 3.86 (6 H, s), 4.50 and 4.77 (2 H, br), 5.12 (2 H, s), 6.44 and 6.65 (4 H, br), 7.25–7.44 ppm (5 H, m); LC–MS (ES+): *m*/*z* 496.26 ([*M*+Na]^+^, 100 %), 464.28 [*M*+H]^+^; HRMS (ES+): *m*/*z* found 464.2060; C_27_H_30_NO_6_^+^ [*M*+H]^+^ requires 464.2068.

**6-Benzyloxy-2-(2-methoxybenzoyl)-7-methoxy-3,4-dihydroisoquinolin-1(2*H*)-one (15 a)**: Compound **14 a** (400 mg, 1.0 mmol), KMnO_4_ (0.79 g, 5.0 mmol) and 18-crown-6 (50 mg, 0.19 mmol) were mixed in dichloromethane (50 mL) and the reaction mixture was stirred at RT for 8 h. The reaction mixture was diluted with CHCl_3_ and sodium metabisulfite (sat.) was added. The mixture was washed with H_2_O and brine, dried with MgSO_4_, filtered and concentrated in vacuo. Flash column chromatography (hexane/EtOAc 3:1) afforded compound **15 a** as a white powder (170 mg, 40 %), mp: 163–164 °C. ^1^H NMR (270 MHz, CDCl_3_): *δ*=2.98 (2 H, t, *J*=6.2 Hz), 3.63 (3 H, s), 3.83 (3 H, s), 4.19 (2 H, t, *J*=6.2 Hz), 5.22 (2 H, s), 6.72 (1 H, s), 6.86 (1 H, d, *J*=8.4 Hz), 7.01 (1 H, dt, *J*=8.4, 0.8 Hz), 7.28–7.46 (7 H, m), 7.56 ppm (1 H, s); LC–MS (ES+): *m*/*z* 440.49 [*M*+Na]^+^; LC–MS (ES−): *m*/*z* 416.44 [*M*−H]^−^; HRMS (ES+): *m*/*z* found 418.1635; C_25_H_24_NO_5_^+^ [*M*+H]^+^ requires 418.1649.

**6-Benzyloxy-7-methoxy-2-(3-methoxybenzoyl)-3,4-dihydroisoquinolin-1(2*H*)-one (15 b)**: Method as for **15 a** using compound **14 b** (560 mg, 1.38 mmol), KMnO_4_ (1.1 g, 6.9 mmol) and 18-crown-6 (70 mg, 0.26 mmol) in dichloromethane (50 mL) at RT for 16 h. Flash column chromatography (hexane/EtOAc 3:1) afforded compound **15 b** as a white powder (235 mg, 41 %), mp: 141–143 °C. ^1^H NMR (270 MHz, CDCl_3_): *δ*=3.03 (2 H, t, *J*=6.2 Hz), 3.81 (3 H, s), 3.85 (3 H, s), 4.08 (2 H, t, *J*=6.2 Hz), 5.23 (2 H, s), 6.73 (1 H, s), 7.01 (1 H, ddd, *J*=8.2, 2.7, 1.2 Hz), 7.11–7.15 (2 H, m), 7.26–7.45 (6 H, m), 7.56 ppm (1 H, s); LC–MS (ES+): *m*/*z* 440.43 [*M*+Na]^+^; LC–MS (ES−): *m*/*z* 416.38 [*M*−H]^−^; HRMS (ES+): *m*/*z* found 418.1636; C_25_H_24_NO_5_^+^ [*M*+H]^+^ requires 418.1649.

**6-Benzyloxy-2-(3-cyanobenzoyl)-7-methoxy-3,4-dihydroisoquinolin-1(2*H*)-one (15 c)**: Method as for **15 a** using compound **14 c** (315 mg, 0.79 mmol), KMnO_4_ (0.62 g, 3.8 mmol) and 18-crown-6 (40 mg, 0.15 mmol) in dichloromethane (40 mL) at RT for 16 h. Flash column chromatography (hexane/EtOAc 3:2) afforded compound **15 c** as a white powder (100 mg, 31 %), mp: 195–196 °C. ^1^H NMR (270 MHz, CDCl_3_): *δ*=3.05 (2 H, t, *J*=6.2 Hz), 3.82 (3 H, s), 3.86 (2 H, t, *J*=6.2 Hz), 5.24 (2 H, s), 6.74 (1 H, s, 7.30–7.45 (5 H, m), 7.49–7.55 (2 H, m), 7.74 (1 H, ddd, *J*=7.9, 1.7, 1.5 Hz), 7.79 (1 H, ddd, *J*=7.9, 1.7, 1.2 Hz), 7.82 ppm (1 H, d, *J*=1.5, 1.2 Hz); LC–MS (ES+): *m*/*z* 435.58 [*M*+Na]^+^; HRMS (ES+): *m*/*z* found 413.1496; C_25_H_21_N_2_O_4_^+^ [*M*+H]^+^ requires 413.1496.

**6-Benzyloxy-2-(4-methoxybenzoyl)-7-methoxy-3,4-dihydroisoquinolin-1(2*H*)-one (15 d)**: Method as for **15 a** using compound **14 d** (403 mg, 1.0 mmol), KMnO_4_ (0.79 g, 5.0 mmol) and 18-crown-6 (53 mg, 0.2 mmol) in dichloromethane (50 mL) at RT for 16 h. Flash column chromatography (hexane/EtOAc 4:1 to 1:1 to EtOAc) afforded compound **15 d** as a white powder (188 mg, 45 %), mp: 151–152 °C. ^1^H NMR (270 MHz, CDCl_3_): *δ*=3.02 (2 H, t, *J*=6.1 Hz), 3.83 (3 H, s), 3.86 (3 H, s), 4.04 (2 H, t, *J*=6.1 Hz), 5.23 (2 H, s), 6.73 (1 H, s), 6.88 (2 H, d, *J*=8.7 Hz), 7.30–7.46 (5 H, m), 7.58 (1 H, s), 7.62 ppm (2 H, d, *J*=8.7 Hz); HRMS (ES+): *m*/*z* found 418.1651; C_25_H_24_NO_5_^+^ [*M*+H]^+^ requires 418.1649.

**6-Benzyloxy-2-(3,4-dimethoxybenzoyl)-7-methoxy-3,4-dihydroisoquinolin-1(2*H*)-one (15 e)**: Method as for **15 a** using compound **14 e** (450 mg, 1.04 mmol), KMnO_4_ (0.82 g, 5.2 mmol) and 18-crown-6 (50 mg, 0.19 mmol) in dichloromethane (50 mL) at RT for 8 h. Flash column chromatography (hexane/EtOAc 3:1) afforded compound **15 e** as a white powder (170 mg, 37 %), mp: 200–201 °C. ^1^H NMR (270 MHz, CDCl_3_): *δ*=3.04 (2 H, t, *J*=6.2 Hz), 3.87 (3 H, s), 3.90 (6 H, s), 4.04 (2 H, t, *J*=6.2 Hz), 5.23 (2 H, s), 6.74 (1 H, s), 6.81 (1 H, d, *J*=8.4 Hz), 7.20 (1 H, dd, *J*=8.4, 2.0 Hz), 7.26 (1 H, d, *J*=2.0 Hz), 7.28–7.46 (5 H, m), 7.58 ppm (1 H, s); LC–MS (ES+): *m*/*z* 470.52 [*M*+Na]^+^; LC–MS (ES−): *m*/*z* 446.47 [*M*−H]^−^.

**6-Benzyloxy-2-(3,5-dimethoxybenzoyl)-7-methoxy-3,4-dihydroisoquinolin-1(2*H*)-one (15 f)**: Method as for **15 a** using compound **14 f** (433 mg, 1.0 mmol), KMnO_4_ (0.79 g, 5.0 mmol) and 18-crown-6 (53 mg, 0.2 mmol) in dichloromethane (50 mL) at RT for 16 h. Flash column chromatography (hexane/EtOAc 10:1 to 4:1) afforded compound **15 f** as a white powder (193 mg, 43 %), mp: 180–181 °C. ^1^H NMR (270 MHz, CDCl_3_): *δ*=3.02 (2 H, t, *J*=6.2 Hz), 3.78 (6 H, s), 3.85 (3 H, s), 4.06 (2 H, t, *J*=6.2 Hz), 5.23 (2 H, s), 6.56 (1 H, t, *J*=2.2 Hz), 6.70 (2 H, d, *J*=2.2 Hz), 6.73 (1 H, s), 7.29–7.46 (5 H, m), 7.56 ppm (1 H, s); LC–MS (APCI+): *m*/*z* 448.54 [*M*+H]^+^; HRMS (ES+): *m*/*z* found 448.1756; C_26_H_26_NO_6_^+^ [*M*+H]^+^ requires 448.1755.

**6-Benzyloxy-7-methoxy-2-(3,4,5-trimethoxybenzoyl)-3,4-dihydroisoquinolin-1(2*H*)-one (15 g)**: Method as for **15 a** using compound **14 g** (0.85 g, 1.83 mmol) and 18-crown-6 (48 mg, 0.18 mmol) in CHCl_3_ (30 mL) at 0 °C for 2 h then at RT 16 h. The reaction mixture was diluted with CHCl_3_ and sodium bisulfite (sat.) and HCl (2 m, 1.0 mL, 2.0 mmol) were added. The mixture was washed with H_2_O and brine, dried with MgSO_4_, filtered and concentrated in vacuo. Flash column chromatography (hexane/EtOAc 3:1 to 1:1) afforded compound **15 g** as a white powder (220 mg, 25 %), mp: 195–196 °C. ^1^H NMR (270 MHz, CDCl_3_): *δ*=3.05 (2 H, t, *J*=6.2 Hz), 3.83 (6 H, s), 3.87 (3 H, s), 3.87 (3 H, s), 4.05 (2 H, t, *J*=6.2 Hz), 5.23 (2 H, s), 6.75 (1 H, s), 6.84 (2 H, s), 7.29–7.46 (5 H, m), 7.56 ppm (1 H, s); LC–MS (ES+): *m*/*z* 500.18 ([*M*+Na]^+^, 100 %), 478.20 [*M*+H]^+^; HRMS (ES+): *m*/*z* found 478.1844; C_27_H_28_NO_7_^+^ [*M*+H]^+^ requires 478.1860.

**6-Hydroxy-2-(2-methoxybenzoyl)-7-methoxy-3,4-dihydroisoquinolin-1(2*H*)-one (16 a)**: Method as for **8 a** using compound **15 a** (145 mg, 0.35 mmol) and Pd/C (10 %, 20 mg) in THF (10 mL) and MeOH (10 mL) under hydrogen at RT for 2 h. The residue was stirred in EtOAc (5 mL), filtered and dried in vacuo to afford compound **16 a** as a white powder (105 mg, 92 %), mp: 199–200 °C. ^1^H NMR (270 MHz, [D_6_]DMSO): *δ*=2.94 (2 H, t, *J*=6.2 Hz), 3.57 (3 H, s), 3.74 (3 H, s), 4.05 (2 H, t, *J*=6.2 Hz), 6.76 (1 H, s), 6.93–7.00 (2 H, m), 7.27 (1 H, dd, *J*=7.4, 1.7 Hz), 7.32 (1 H, s), 7.34–7.41 (1 H, m), 10.21 ppm (1 H, s); LC–MS (ES−): *m*/*z* 326.51 [*M*−H]^−^; HRMS (ES+): *m*/*z* found 328.1167; C_18_H_18_NO_5_^+^ [*M*+H]^+^ requires 328.1179.

**6-Hydroxy-2-(3-methoxybenzoyl)-7-methoxy-3,4-dihydroisoquinolin-1(2*H*)-one (16 b)**: Method as for **8 a** using compound **15 b** (200 mg, 0.48 mmol) and Pd/C (10 %, 25 mg) in THF (10 mL) and MeOH (10 mL) under hydrogen at RT for 1 h. The residue was stirred in EtOAc (5 mL), filtered and dried in vacuo to afford compound **16 b** as a white powder (125 mg, 80 %), mp: 198–199 °C. ^1^H NMR (270 MHz, [D_6_]DMSO): *δ*=3.03 (2 H, t, *J*=5.9 Hz), 3.75 (3 H, s), 3.76 (3 H, s), 3.95 (2 H, t, *J*=5.9 Hz), 6.77 (1 H, s), 7.05–7.10 (3 H, m), 7.32 (1 H, dt, *J*=6.7, 1.0 Hz), 7.34 (1 H, s), 10.21 ppm (1 H, s); LC–MS (ES−): *m*/*z* 326.58 [*M*−H]^−^; HRMS (ES+): *m*/*z* found 328.1168; C_18_H_18_NO_5_^+^ [*M*+H]^+^ requires 328.1179.

**2-(3-Cyanobenzoyl)-6-hydroxy-7-methoxy-3,4-dihydroisoquinolin-1(2*H*)-one (16 c)**: Method as for **8 a** using compound **15 c** (155 mg, 0.38 mmol) and Pd/C (10 %, 20 mg) in THF (10 mL) and MeOH (10 mL) under hydrogen at RT for 1 h. The residue was dissolved in hot EtOAc (5 mL), filtered and concentrated then stirred in Et_2_O (20 mL), filtered and dried in vacuo to afford compound **16 c** as a white powder (71 mg, 59 %), mp: 197–198 °C. ^1^H NMR (270 MHz, [D_6_]acetone): *δ*=3.14 (2 H, t, *J*=6.2 Hz), 3.85 (3 H, s), 4.08 (2 H, t, *J*=6.2 Hz), 6.83 (1 H, s), 7.42 (1 H, s), 7.65 (1 H, dd, *J*=8.2, 7.6 Hz), 7.86–7.91 (2 H, m), 8.00–8.06 (1 H, m), 8.71 ppm (1 H, s, br); LC–MS (ES−): *m*/*z* 321.47 (M^+^-H); HRMS (ES+): *m*/*z* found 323.1012; C_18_H_15_N_2_O_4_^+^ [*M*+H]^+^ requires 323.1026.

**6-Hydroxy-2-(4-methoxybenzoyl)-7-methoxy-3,4-dihydroisoquinolin-1(2*H*)-one (16 d)**: Method as for **8 a** using compound **15 d** (96 mg, 0.23 mmol) and Pd/C (10 %, 20 mg) in THF (10 mL) and EtOH (10 mL) under hydrogen at RT for 0.5 h. The residue was stirred in EtOAc (10 mL) and hexane (2 mL), filtered and dried in vacuo to afford compound **16 d** as a white powder (72 mg, 96 %), mp: 224–225 °C. ^1^H NMR (270 MHz, [D_6_]DMSO): *δ*=3.01 (2 H, t, *J*=5.7 Hz), 3.77 (3 H, s), 3.82 (3 H, s), 3.90 (2 H, t, *J*=5.7 Hz), 6.77 (1 H, s), 6.95 (2 H, d, *J*=8.6 Hz), 7.36 (1 H, s), 7.55 (2 H, d, *J*=8.6 Hz), 10.19 ppm (1 H, s, br); LC–MS (APCI+): *m*/*z* 328.46 [*M*+H]^+^; HRMS (ES+): *m*/*z* found 328.1181; C_18_H_18_NO_5_^+^ [*M*+H]^+^ requires 328.1179.

**6-Hydroxy-2-(3,4-dimethoxybenzoyl)-7-methoxy-3,4-dihydroisoquinolin-1(2*H*)-one (16 e)**: Method as for **8 a** using compound **15 e** (165 mg, 0.37 mmol) and Pd/C (10 %, 20 mg) in THF (150 mL) under hydrogen at RT for 2 h. Crystallisation from EtOAc (5 mL) afforded compound **16 e** as a white powder (115 mg, 87 %), mp: 199–200 °C. ^1^H NMR (400 MHz, [D_6_]DMSO): *δ*=3.02 (2 H, t, *J*=5.9 Hz), 3.75 (3 H, s), 3.76 (3 H, s), 3.81 (3 H, s), 3.90 (2 H, t, *J*=5.9 Hz), 6.77 (1 H, s), 6.96 (1 H, d, *J*=9.2 Hz), 7.15–7.18 (2 H, m), 7.36 (1 H, s), 10.20 ppm (1 H, s, br); LC–MS (ES−): *m*/*z* 356.60 [*M*−H]^−^; HRMS (ES+): *m*/*z* found 358.1273; C_19_H_20_NO_6_^+^ [*M*+H]^+^ requires 358.1285.

**6-Hydroxy-2-(3,5-dimethoxybenzoyl)-7-methoxy-3,4-dihydroisoquinolin-1(2*H*)-one (16 f)**: Method as for **8 a** using compound **15 f** (200 mg, 0.45 mmol) and Pd/C (10 %, 30 mg) in THF (15 mL) and MeOH (15 mL) under hydrogen at RT for 3 h. The residue was stirred in Et_2_O, filtered and dried in vacuo to afford compound **16 f** as a white powder (130 mg, 81 %), mp: 198–199 °C. ^1^H NMR (270 MHz, CDCl_3_): *δ*=3.05 (2 H, t, *J*=6.2 Hz), 3.78 (6 H, s), 3.86 (3 H, s), 4.07 (2 H, t, *J*=6.2 Hz), 6.16 (1 H, s), 6.56 (1 H, t, *J*=2.2 Hz), 6.71 (2 H, d, *J*=2.2 Hz), 6.79 (1 H, s), 7.54 ppm (1 H, s); LC–MS (APCI+): *m*/*z* 358.25 [*M*+H]^+^; HRMS (ES+): *m*/*z* found 358.1285; C_19_H_20_NO_6_^+^ [*M*+H]^+^ requires 358.1285.

**6-Hydroxy-7-methoxy-2-(3,4,5-trimethoxybenzoyl)-3,4-dihydroisoquinolin-1(2*H*)-one (16 g)**: Method as for **8 a** using compound **15 g** (180 mg, 0.38 mmol) and Pd/C (10 %, 40 mg) in THF (20 mL) and MeOH (20 mL) under hydrogen at RT for 24 h. The residue was stirred in Et_2_O, filtered, washed with Et_2_O and dried in vacuo to afford compound **16 g** as a white powder (140 mg, 96 %), mp: 175–176 °C. ^1^H NMR (270 MHz, CDCl_3_): *δ*=3.07 (2 H, t, *J*=6.4 Hz), 3.83 (6 H, s), 3.88 (3 H, s), 3.89 (3 H, s), 4.06 (2 H, t, *J*=6.4 Hz), 6.18 (1 H, s), 6.80 (1 H, s), 6.85 (2 H, s), 7.55 ppm (1 H, s); HRMS (ES+): *m*/*z* found 388.1380; C_20_H_22_NO_7_^+^ [*M*+H]^+^ requires 388.1391; LC–MS (ES−): *m*/*z* 386.24 [*M*−H]^−^.

**2-(2-Methoxybenzoyl)-7-methoxy-6-sulfamoyloxy-3,4-dihydroisoquinolin-1(2*H*)-one (17 a)**: Method as for **9 a** using compound **16 a** (70 mg, 0.21 mmol) and sulfamoyl chloride (0.43 mmol) in DMA (1.0 mL) at RT for 16 h. The residue was stirred in Et_2_O (30 mL), filtered and dried in vacuo to afford compound **17 a** as a white powder (65 mg, 75 %), mp: 165–166 °C. ^1^H NMR (400 MHz, [D_6_]DMSO): *δ*=3.05 (2 H, t, *J*=6.2 Hz), 3.60 (3 H, s), 3.80 (3 H, s), 4.11 (2 H, t, *J*=6.2 Hz), 6.96–7.03 (2 H, m), 7.31 (1 H, dd, *J*=7.7, 1.7 Hz), 7.39 (1 H, s), 7.41 (1 H, dt, *J*=8.4, 1.7 Hz), 7.53 (1 H, s), 8.18 ppm (2 H, s, br); LC–MS (ES−): *m*/*z* 405.50 [*M*−H]^−^; HRMS (ES+): *m*/*z* found 407.0898; C_18_H_19_N_2_O_7_S^+^ [*M*+H]^+^ requires 407.0907.

**2-(3-Methoxybenzoyl)-7-methoxy-6-sulfamoyloxy-3,4-dihydroisoquinolin-1(2*H*)-one (17 b)**: Method as for **9 a** using compound **16 b** (80 mg, 0.24 mmol) and sulfamoyl chloride (0.48 mmol) in DMA (1.0 mL) at RT for 16 h. The residue was stirred in Et_2_O (30 mL), filtered and dried in vacuo to afford compound **17 b** as a white powder (75 mg, 77 %), mp: 173–174 °C. ^1^H NMR (400 MHz, [D_6_]DMSO): *δ*=3.15 (2 H, t, *J*=5.9 Hz), 3.77 (3 H, s), 3.81 (3 H, s), 4.01 (2 H, t, *J*=5.9 Hz), 7.08–7.18 (3 H, m), 7.31–7.36 (1 H, m), 7.41 (1 H, s), 7.57 (1 H, s), 8.18 ppm (2 H, s, br); LC–MS (ES−): *m*/*z* 405.43 [*M*−H]^−^; HRMS (ES+): *m*/*z* found 407.0895; C_18_H_19_N_2_O_7_S^+^ [*M*+H]^+^ requires 407.0907.

**2-(3-Cyanobenzoyl)-7-methoxy-6-sulfamoyloxy-3,4-dihydroisoquinolin-1(2*H*)-one (17 c)**: Method as for **9 a** using compound **16 c** (50 mg, 0.16 mmol) and sulfamoyl chloride (0.40 mmol) in DMA (0.5 mL) at RT for 16 h. Crystallisation from EtOAc afforded compound **17 c** as a white powder (45 mg, 70 %), mp: 171–172 °C. ^1^H NMR (270 MHz, [D_6_]acetone): *δ*=3.23 (2 H, t, *J*=6.2 Hz), 3.88 (3 H, s), 4.14 (2 H, t, *J*=6.2 Hz), 7.24 (2 H, s, br), 7.40 (1 H, s), 7.58 (1 H, s), 7.69 (1 H, dt, *J*=7.9, 0.8 Hz), 7.91–7.98 (2 H, m), 8.06 ppm (1 H, dt, *J*=1.8, 0.8 Hz); LC–MS (ES−): *m*/*z* 400.53 [*M*−H]^−^; HRMS (ES+): *m*/*z* found 402.0739; C_18_H_16_N_3_O_6_S^+^ [*M*+H]^+^ requires 402.0754.

**2-(4-Methoxybenzoyl)-7-methoxy-6-sulfamoyloxy-3,4-dihydroisoquinolin-1(2*H*)-one (17 d)**: Method as for **9 a** using compound **16 d** (90 mg, 0.28 mmol) and sulfamoyl chloride (0.55 mmol) in DMA (1.0 mL) at RT for 18 h. Flash column chromatography (hexane/EtOAc 10:1 to 4:1) afforded compound **17 d** as a white powder (80 mg, 71 %), mp: 171–172 °C. ^1^H NMR (270 MHz, CDCl_3_/[D_4_]MeOH 5:1): *δ*=3.08 (2 H, s, br), 3.10 (2 H, t, *J*=6.4 Hz), 3.83 (3 H, s), 3.85 (3 H, s), 4.03 (2 H, t, *J*=6.4 Hz), 6.88 (2 H, dt, *J*=8.9, 2.5 Hz), 7.31 (1 H, s), 7.61 (2 H, dt, *J*=8.9, 2.5 Hz), 7.67 ppm (1 H, s); LC–MS (APCI+): *m*/*z* 407.42 [*M*+H]^+^; HRMS (ES+): *m*/*z* found 407.0904; C_18_H_19_N_2_O_7_S^+^ [*M*+H]^+^ requires 407.0907.

**2-(3,4-Dimethoxybenzoyl)-7-methoxy-6-sulfamoyloxy-3,4-dihydroisoquinolin-1(2*H*)-one (17 e)**: Method as for **9 a** using compound **16 e** (71 mg, 0.20 mmol) and sulfamoyl chloride (0.40 mmol) in DMA (1.0 mL) at RT for 16 h. After addition of H_2_O (5 mL) the reaction mixture was extracted into EtOAc (2×50 mL), the organic layers washed with H_2_O and brine, then dried (MgSO_4_) and evaporated. The residue was stirred in EtOAc (5 mL), filtered and dried in vacuo to afford compound **17 e** as a white powder (55 mg, 77 %), mp: 180–181 °C. ^1^H NMR (270 MHz, ([D_6_]acetone): *δ*=3.19 (2 H, t, *J*=6.2 Hz), 3.81 (3 H, s), 3.87 (3 H, s), 3.88 (3 H, s), 4.03 (2 H, t, *J*=6.2 Hz), 6.96 (1 H, d, *J*=8.9 Hz), 7.21 (2 H, s, br), 7.28 (1 H, s), 7.30 (1 H, d, *J*=8.9 Hz), 7.39 (1 H, s), 7.62 ppm (1 H, s); LC–MS (ES−): *m*/*z* 435.44 [*M*−H]^−^; HRMS (ES+): *m*/*z* found 437.1011; C_19_H_21_N_2_O_8_S^+^ [*M*+H]^+^ requires 437.1013.

**2-(3,5-Dimethoxybenzoyl)-7-methoxy-6-sulfamoyloxy-3,4-dihydroisoquinolin-1(2*H*)-one (17 f)**: Method as for **9 a** using compound **16 f** (80 mg, 0.22 mmol) and sulfamoyl chloride (0.45 mmol) in DMA (1.0 mL) at RT for 18 h. The residue was stirred in Et_2_O, filtered and dried in vacuo to afford compound **17 f** as a white powder (76 mg, 78 %), mp: 169–170 °C. ^1^H NMR (270 MHz, (CDCl_3_/[D_4_]MeOH 10:1): *δ*=2.46 (2 H, s, br), 3.08 (2 H, t, *J*=6.2 Hz), 3.75 (6 H, s), 3.82 (3 H, s), 4.05 (2 H, t, *J*=6.2 Hz), 6.56 (1 H, t, *J*=2.3 Hz), 6.68 (2 H, d, *J*=2.3 Hz), 7.29 (1 H, s), 7.65 ppm (1 H, s); LC–MS (APCI+): *m*/*z* 437.39 [*M*+H]^+^; HRMS (ES+) *m*/*z* found 437.1011; C_19_H_21_N_2_O_8_S^+^ [*M*+H]^+^ requires 437.1013.

**7-Methoxy-2-(3,4,5-trimethoxybenzoyl)-6-sulfamoyloxy-3,4-dihydroisoquinolin-1(2*H*)-one (17 g)**: Method as for **9 a** using compound **16 g** (80 mg, 0.21 mmol) and sulfamoyl chloride (0.6 mmol) in DMA (1.0 mL) at RT for 24 h. H_2_O (20 mL) was added and the mixture extracted with EtOAc (50 mL) and THF (50 mL). The organic layer was washed with H_2_O and brine, dried (MgSO_4_), filtered and concentrated in vacuo. The residue was stirred in Et_2_O (5 mL) and EtOAc (5 mL), filtered and dried to afford compound **17 g** as a white powder (80 mg, 82 %), mp: 196–197 °C. ^1^H NMR (270 MHz, [D_6_]DMSO): *δ*=3.16 (2 H, t, *J*=5.7 Hz), 3.72 (3 H, s), 3.75 (6 H, s), 3.81 (3 H, s), 3.98 (2 H, t, *J*=5.7 Hz), 6.90 (2 H, s), 7.40 (1 H, s), 7.57 (1 H, s), 8.18 ppm (2 H, s, br); LC–MS (ES−): *m*/*z* 465.22 [*M*−H]^−^; HRMS (ES+): *m*/*z* found 467.1109; C_20_H_23_N_2_O_9_S^+^ [*M*+H]^+^ requires 467.1119.

**2-(3,4-Dimethoxybenzoyl)-6-hydroxy-7-methoxy-1,2,3,4-tetrahydroisoquinoline (18 a)**: Method as for **8 a** using **14 e** (403 mg, 0.93 mmol) and Pd/C (10 %, 40 mg) in THF (15 mL) and MeOH (15 mL) under hydrogen at RT for 1.5 h. The resulting solid was stirred in Et_2_O, filtered and dried in vacuo to afford compound **18 a** as a white solid (300 mg, 94 %), mp: 132–133 °C. ^1^H NMR (270 MHz, CDCl_3_): *δ*=2.80 (2 H, br), 3.83 (2 H, br), 3.88 (3 H, s), 3.90 (6 H, s), 4.61 and 4.72 (2 H, br), 5.62 (1 H, s), 6.60 (1 H, br), 6.69 (1 H, br), 6.86 (1 H, d, *J*=8.9 Hz), 7.02 (1 H, s), 7.03 (1 H, d, *J*=8.9 Hz), 8.84 ppm (1 H, s); LC–MS (APCI+): *m*/*z* 344.27 [*M*+H]^+^; HRMS (ES+): *m*/*z* found 344.1493; C_19_H_22_NO_5_^+^ [*M*+H]^+^ requires 344.1492.

**2-(3,5-Dimethoxybenzoyl)-6-hydroxy-7-methoxy-1,2,3,4-tetrahydroisoquinoline (18 b)**: Method as for **8 a** using **14 f** (390 mg, 0.90 mmol) and Pd/C (10 %, 40 mg) in THF (15 mL) and MeOH (15 mL) under hydrogen at RT for 2 h. The resulting solid was stirred in Et_2_O, filtered and dried in vacuo to afford compound **18 b** as a white solid (290 mg, 94 %), mp: 136–137 °C. ^1^H NMR (270 MHz, CDCl_3_): *δ*=2.73 and 2.84 (2 H, br), 3.59 and 3.93 (2 H, br), 3.79 (9 H, s), 4.48 and 4.77 (2 H, br), 5.58 (1 H, s), 6.38 and 6.64 (1 H, br), 6.50 (1 H, t, *J*=2.2 Hz), 6.55 (2 H, d, *J*=2.2 Hz), 6.68 ppm (1 H, br); LC–MS (APCI+): *m*/*z* 344.33 [*M*+H]^+^; HRMS (ES+): *m*/*z* found 344.1493; C_19_H_22_NO_5_^+^ [*M*+H]^+^ requires 344.1492.

**2-(3,4-Dimethoxybenzoyl)-7-methoxy-6-sulfamoyloxy-1,2,3,4-tetrahydroisoquinoline (19 a)**: Method as for **9 a** using **18 a** (75 mg, 0.24 mmol) and sulfamoyl chloride (0.48 mmol) in anhydrous DMA (1.0 mL) at RT for 24 h. The residue was stirred in Et_2_O, filtered and dried in vacuo to afford compound **19 a** as a white powder (80 mg, 85 %), mp: 155–156 °C. ^1^H NMR (270 MHz, CDCl_3_/[D_4_]MeOH 5:1): *δ*=2.73 (2 H, br), 3.59 and 3.72 (2 H, br), 3.77 (3 H, s), 3.79 (6 H, s), 4.52 and 4.65 (2 H, br), 6.61 and 6.70 (1 H, br), 6.80 (1 H, d, *J*=8.2 Hz), 6.87 (1 H, d, *J*=2.0 Hz), 6.90 (1 H, dd, *J*=8.2, 2.0 Hz), 7.03 ppm (1 H, s); LC–MS (APCI+): *m*/*z* 423.42 [*M*+H]^+^; HRMS (ES+): *m*/*z* found 423.1220; C_19_H_23_N_2_O_7_S^+^ [*M*+H]^+^ requires 423.1220.

**2-(3,5-Dimethoxybenzoyl)-7-methoxy-6-sulfamoyloxy-1,2,3,4-tetrahydroisoquinoline (19 b)**: Method as for **9 a** using **18 b** (75 mg, 0.24 mmol) and sulfamoyl chloride (0.48 mmol) in anhydrous DMA (1.0 mL) at RT for 24 h. The residue was stirred in Et_2_O, filtered and dried in vacuo to afford compound **19 b** as a white powder (80 mg, 85 %), mp: 170–171 °C. ^1^H NMR (270 MHz, CDCl_3_/[D_4_]MeOH 5:1): *δ*=2.70 and 2.80 (2 H, t, *J*=5.3 Hz), 3.55 and 3.82 (2 H, t, *J*=5.3 Hz), 3.63 (2 H, s), 3.71 (9 H, s), 4.46 and 4.72 (2 H, br), 6.44 (2 H, s), 6.46 and 6.74 (1 H, br), 7.05 ppm (1 H, br); LC–MS (APCI+): *m*/*z* 423.35 [*M*+H]^+^; HRMS (ES+): *m*/*z* found 423.1224; C_19_H_23_N_2_O_7_S^+^ [*M*+H]^+^ requires 423.1220.

## Supporting Information

As a service to our authors and readers, this journal provides supporting information supplied by the authors. Such materials are peer reviewed and may be re-organized for online delivery, but are not copy-edited or typeset. Technical support issues arising from supporting information (other than missing files) should be addressed to the authors.

miscellaneous_information

## References

[1] Bubert C, Leese MP, Mahon MF, Ferrandis E, Regis-Lydi S, Kasprzyk PG, Newman SP, Ho YT, Purohit A, Reed MJ, Potter BVL (2007). J. Med. Chem.

[2] Jourdan FL, Leese MP, Dohle W, Ferrandis E, Newman SP, Chander S, Purohit A, Potter BVL (2011). J. Med. Chem.

[3] Jourdan FL, Leese MP, Dohle W, Hamel E, Ferrandis E, Newman SP, Purohit A, Reed MJ, Potter BVL (2010). J. Med. Chem.

[4] Leese MP, Hejaz HAM, Mahon MF, Newman SP, Purohit A, Reed MJ, Potter BVL (2005). J. Med. Chem.

[5] Leese MP, Jourdan FL, Gaukroger K, Mahon MF, Newman SP, Foster PA, Stengel C, Regis-Lydi S, Ferrandis E, Di Fiore A, De Simone G, Supuran CT, Purohit A, Reed MJ, Potter BVL (2008). J. Med. Chem.

[6] Leese MP, Leblond B, Smith A, Newman SP, Di Fiore A, De Simone G, Supuran CT, Purohit A, Reed MJ, Potter BVL (2006). J. Med. Chem.

[7] Leese MP, Jourdan FL, Dohle W, Kimberley MR, Thomas MP, Bai R, Hamel E, Ferrandis E, Potter BVL (2012). ACS Med. Chem. Lett.

[8] Leese MP, Jourdan FL, Major MR, Dohle W, Hamel E, Ferrandis E, Fiore A, Kasprzyk PG, Potter BVL (2014). ChemMedChem.

[9] Dohle W, Leese MP, Jourdan F, Major MR, Bai R, Hamel E, Ferrandis E, Kasprzyk PG, Fiore A, Newman SP, Purohit A, Potter BVL (2014). ChemMedChem.

[10] Leese MP, Jourdan F, Kimberley MR, Cozier GE, Thiyagarajan N, Stengel C, Regis-Lydi S, Foster PA, Newman SP, Acharya KR, Ferrandis E, Purohit A, Reed MJ, Potter BVL (2010). Chem. Commun.

[11] Dohle W, Jourdan F, Chapman CJ, Leese MP, Hamel E, Ferrandis E, Potter BVL (2014). ChemMedChem.

[12] Okada M, Iwashita S, Koizumi N (2000). Tetrahedron Lett.

[13a] Ho YT, Purohit A, Vicker N, Newman SP, Robinson JJ, Leese MP, Ganeshapillai D, Woo LWL, Potter BVL, Reed MJ (2003). Biochem. Biophys. Res. Commun.

[13b] Newman SP, Ireson CR, Tutill HJ, Day JM, Parsons MFC, Leese MP, Potter BVL, Reed MJ, Purohit A (2006). Cancer Res.

[13c] Ireson CR, Chander SK, Purohit A, Perera S, Newman SP, Parish D, Leese MP, Smith AC, Potter BVL, Reed MJ (2004). Br. J. Cancer.

[14] Newman SP, Leese MP, Purohit A, James DRC, Rennie CE, Potter BVL, Reed MJ (2004). Int. J. Cancer.

[15] http://www.ccdc.cam.ac.uk/data_request/cif.

[16] Hamel E, Lin CM (1984). Biochemistry.

[17] Hamel E (2003). Cell Biochem. Biophys.

[18] Verdier-Pinard P, Lai JY, Yoo HD, Yu J, Marquez B, Nagle DG, Nambu M, White JD, Falck JR, Gerwick WH, Day BW, Hamel E (1998). Mol. Pharmacol.

[19] Lin CM, Ho HH, Pettit GR, Hamel E (1989). Biochemistry.

[20] Appel R, Berger G (1958). Chem. Ber.

[21] Woo LWL, Lightowler M, Purohit A, Reed MJ, Potter BVL (1996). J. Steroid Biochem. Mol. Biol.

[22] Sheldrick GM (1990). Acta Crystallogr. Sect. A.

