# Supplementary material for: Synthesis, Anti-tubulin and Antiproliferative SAR of Steroidomimetic Dihydroisoquinolinones
Source: ChemMedChem. 2014 Mar 5;9(4):798–812. doi: 10.1002/cmdc.201400017 (PMC4114533; doi:10.1002/cmdc.201400017)
Supplement: Supplementary file 1 — miscellaneous_information [file cmdc-9-798-sd1.pdf]

CHEMMEDCHEM
Chemistry & Drug Discovery

Supporting Information

© Copyright Wiley-VCH Verlag GmbH & Co. KGaA, 69451 Weinheim, 2014

Synthesis, Anti-tubulin and Antiproliferative SAR of Steroidomimetic Dihydroisoquinolinones

Mathew P. Leese,[a] Fabrice L. Jourdan,[a] Meriel R. Major,[a] Wolfgang Dohle,[a] Mark P. Thomas,[a] Ernest Hamel,[b] Eric Ferrandis,[c] Mary F. Mahon,[d] Simon P. Newman,[e] Atul Purohit,[e] and Barry V. L. Potter*[a]

cmdc_201400017_sm_miscellaneous_information.pdf

Contents:

^{13}C NMR Data **Pages 1-4**

Elemental Analyses **Pages 4-6**

Computational Methods **Pages 6-7**

^{13}C NMR data for selected compounds.

4 (67.5 MHz, $CDCl_3$): δ = 25.4, 36.4, 51.3, 104.0, 111.1, 129.5, 146.9, 150.9, 152.5, 206.0.

6a (67.5 MHz, $CDCl_3$): δ = 28.0, 40.5, 56.3, 70.9, 110.6, 111.7, 121.8, 127.3, 128.1, 128.7, 132.4, 136.5, 148.4, 151.4, 166.4.

6c (67.5 MHz, $CDCl_3$): δ = 13.0, 18.0, 27.8, 40.5, 55.6, 111.0, 118.9, 122.1, 132.3, 149.2, 150.0, 166.9.

7a (67.5 MHz, $CDCl_3$): δ = 27.9, 45.4, 46.3, 55.4 56.3, 65.9, 110.2, 110.3, 111.1, 111.2, 120.7, 120.8, 122.3, 124.9, 125.6, 128.2, 128.4, 128.9, 129.4, 131.7, 148.4, 151.1, 156.5, 157.6, 164.8.

7b1 (67.5 MHz, $CDCl_3$): δ = 13.0, 18.0, 27.5, 45.7, 50.4, 55.3, 55.6, 111.5, 112.8, 113.6, 118.5, 120.4, 122.6, 129.6, 131.5, 139.3, 148.9, 150.0, 159.9, 164.6.

7b2 (67.5 MHz, $CDCl_3$): δ = 27.6, 45.6, 50.4, 55.2 56.2, 70.8, 111.5, 112.6, 112.8 113.6, 118.5, 119.4, 120.4, 122.3, 129.7, 129.8, 131.6, 138.2, 139.3, 148.5, 151.0, 159.9, 164.7.

7c (67.5 MHz, $CDCl_3$): δ = 13.0, 18.0, 27.4, 45.6, 49.9, 55.3, 55.6, 111.5, 113.9, 118.5, 122.4, 129.5, 129.9, 131.4, 148.8, 150.0, 158.9, 164.8.

7d (67.5 MHz, $CDCl_3$): δ = 27.8, 45.5, 50.5, 55.5, 56.3, 70.9, 99.3, 106.0, 111.2, 111.5, 122.3, 127.3, 128.1, 128.8, 131.6, 136.6, 140.2, 148.6, 151.0, 161.1, 164.7.

7e (67.5 MHz, $CDCl_3$): δ = 27.8, 45.5, 50.6, 56.3 61.0, 70.9, 105.0, 111.2, 111.5, 122.3, 127.2, 128.1, 128.8, 131.5, 133.5, 136.5, 137.2, 148.6, 151.1, 153.5, 164.7.

8a (67.5 MHz, $CDCl_3$): δ = 27.7, 45.5, 46.3, 55.5, 56.3, 110.3, 110.5, 112.7 120.7 121.9 125.7, 128.4, 129.3, 132.6 145.5, 148.9, 157.6, 164.9.

8b (67.5 MHz, $CDCl_3$): δ = 27.6, 45.6, 50.4, 55.3, 56.3, 110.6, 112.7, 112.9 113.6, 120.4, 121.7, 129.8, 132.5, 139.4, 145.7, 148.9, 159.9, 164.7.

8c (67.5 MHz, $CDCl_3$): δ = 27.5, 45.5, 49.9, 55.3, 56.2, 110.6, 112.8, 114.0, 121.5, 129.4, 129.7, 132.4, 145.8, 149.0, 159.0, 164.8.

8d (67.5 MHz, $CDCl_3$): δ = 27.6, 45.6, 50.5, 55.5, 56.3, 99.3, 105.9, 110.6, 112.7, 121.6, 132.6, 140.2, 145.7, 148.9, 161.1, 164.8.

8e (67.5 MHz, $CDCl_3$): δ = 27.7, 45.5, 50.6, 56.2, 56.3, 61.0, 104.9, 110.5, 112.8, 121.6, 132.5, 133.5, 137.2, 145.7, 149.0, 153.5, 164.8.

9a (67.5 MHz, $CDCl_3$): δ = 27.1, 45.8, 46.1, 55.4, 56.7, 110.4, 113.0, 120.8, 122.8, 125.1, 128.8, 129.4, 129.6, 131.7, 141.1, 150.4, 157.6, 163.6.

9b (67.5 MHz, $CDCl_3$): δ = 27.1, 45.8, 50.7, 55.3, 56.6, 113.0, 113.1, 113.7, 120.3, 122.9, 128.9, 129.8, 131.5, 138.6, 141.2, 150.6, 159.9, 163.6.

9c (67.5 MHz, $CDCl_3$/methanol-d_4 10:1): δ = 27.0, 45.3, 50.2, 55.3, 56.4, 112.8, 114.1, 122.7, 128.6, 129.0, 129.4, 131.3, 141.3, 150.6, 159.1, 163.8.

9d (67.5 MHz, $CDCl_3$): δ = 27.0, 46.0, 50.4, 55.7, 56.5, 99.3, 106.0, 112.4, 122.3, 128.1, 131.7, 140.5, 141.8, 151.0, 161.2, 163.3.

9e (67.5 MHz, DMSO-d_6): δ = 27.0, 45.8, 50.4, 56.4, 56.5, 56.6, 105.3, 112.5, 122.3, 128.2, 131.8, 133.9, 137.1, 141.8, 151.0, 153.5, 163.9.

10a (67.5 MHz, $CDCl_3$): δ = 28.5, 45.3, 56.2, 56.3, 70.9, 111.1, 111.2, 112.1, 120.8, 120.9, 127.3, 127.5, 128.3, 128.8, 132.4, 133.7, 135.4, 136.1, 148.8, 152.5, 156.6, 163.4.

10b (67.5 MHz, $CDCl_3$): δ = 28.5, 45.1, 55.7, 56.1, 70.8, 111.0, 111.1, 113.2, 119.9, 120.3, 120.6, 127.1, 128.2, 128.7, 129.8, 133.4, 135.9, 140.4, 148.8, 152.6, 159.5, 163.3.

10c (67.5 MHz, $CDCl_3$): δ = 28.7, 45.0, 55.7, 56.2, 71.0, 111.1, 111.2, 114.0, 120.9, 127.2, 128.3, 128.8, 130.8, 130.9, 133.5, 136.0, 148.9, 152.6, 163.5, 163.7.

10d (67.5 MHz, $CDCl_3$): δ = 28.7, 45.2, 56.2, 71.0, 111.1, 111.3, 120.4, 126.9, 127.2, 128.3, 128.4, 128.9, 130.1, 133.6, 133.8, 135.0, 135.9, 141.0, 149.0, 152.9, 163.4.

10e (67.5 MHz, $CDCl_3$): δ = 28.3, 44.9, 53.2, 56.2, 70.9, 111.0, 111.3, 120.7, 127.3, 128.3, 128.8, 129.1, 130.6, 132.3, 133.4, 133.7, 134.0, 136.1, 137.9, 148.8, 152.7, 163.5, 167.2.

11a (67.5 MHz, $CDCl_3$): δ = 28.4, 45.3, 56.3, 56.3, 110.7, 112.1, 112.9, 120.4, 120.7, 127.6, 132.4, 134.6, 135.4, 145.9, 150.5, 156.6, 163.4.

11b (67.5 MHz, $CDCl_3$): δ = 28.4, 45.1, 55.7, 56.2, 110.6, 112.8, 113.1, 120.0, 120.2, 120.3, 129.8, 134.4, 140.5, 145.9, 150.6, 159.5, 163.3.

11c (67.5 MHz, $CDCl_3$): δ = 23.4, 44.9, 55.6, 56.2, 110.5, 112.8, 113.9, 120.2, 130.7, 130.8, 134.4, 145.9, 150.5, 163.4, 163.6.

11d (67.5 MHz, $CDCl_3$): δ = 28.5, 45.3, 56.3, 110.6, 113.0, 119.9, 126.9, 128.5, 130.1, 133.8, 134.6, 135.0, 141.0, 146.1, 150.9, 163.4.

11e (67.5 MHz, $CDCl_3$): δ = 28.2, 44.9, 53.2, 56.3, 110.5, 112.9, 120.2, 129.1, 130.5, 132.4, 133.3, 133.7, 134.9, 137.9, 145.9, 150.6, 163.6, 167.3.

12a (67.5 MHz, DMSO-d_6): δ = 27.7, 45.6, 56.6, 57.0, 112.6, 113.6, 120.8, 122.5, 126.5, 127.3, 131.8, 133.5, 136.3, 143.4, 151.3, 157.0, 162.4.

12c (100 MHz, DMSO-d_6): δ = 27.2, 44.8, 55.8, 56.1, 112.0, 114.2, 121.9, 125.8, 130.1, 130.6, 132.9, 142.9, 150.7, 162.1, 163.4.

12d (67.5 MHz, DMSO-d_6): δ = 27.7, 45.5, 56.7, 112.6, 122.5, 126.0, 127.3, 128.2, 131.8, 133.7, 134.1, 134.6, 141.2, 143.6, 151.3, 162.9.

12e (67.5 MHz, DMSO-d_6): δ = 27.5, 45.0, 53.8, 56.6, 112.6, 122.5, 126.2, 129.7, 131.3, 132.7, 133.0, 133.5, 134.8, 137.0, 143.5, 151.3, 162.6, 167.5.

14f (67.5 MHz, $CDCl_3$): δ = 27.8 and 29.0, 40.5 and 49.5, 44.4 and 45.3, 55.5, 56.1, 71.1, 101.9, 104.6, 105.0, 109.2, 109.9, 110.7, 114.2, 125.6, 127.3, 128.0, 128.7, 137.1, 138.1, 146.9, 160.9.

15a (67.5 MHz, $CDCl_3$): δ = 28.3, 43.4, 55.7, 56.2, 70.9, 110.8, 111.2, 111.4, 120.8, 121.1, 127.3, 127.5, 128.3, 128.5, 128.9, 131.5, 134.8, 136.1, 148.7, 152.6, 155.6, 165.0, 170.8.

15b (67.5 MHz, $CDCl_3$): δ = 28.2, 44.6, 55.5, 56.2, 71.0, 111.4, 111.5, 113.2, 117.4, 120.4, 120.7, 127.3, 128.3, 128.9, 129.2, 134.5, 136.1, 138.0, 148.9, 152.8, 159.4, 165.6, 174.5.

15c (67.5 MHz, $CDCl_3$): δ = 28.1, 44.4, 56.1, 71.0, 111.3, 111.4, 112.4, 118.1, 120.0, 127.1, 128.2, 127.8, 129.0, 131.5, 131.9, 134.3, 134.5, 135.8, 138.0, 149.0, 153.1, 165.4, 172.1.

15d (67.5 MHz, $CDCl_3$): δ = 29.7, 44.9, 55.5, 56.2, 70.9, 111.3, 111.5, 113.4, 121.1, 127.3, 128.3, 128.5, 128.9, 130.9, 134.4, 136.1, 148.7, 152.6, 158.3, 162.6, 174.1.

15e (67.5 MHz, $CDCl_3$): δ = 27.5, 44.3, 55.2, 55.3, 55.4, 70.2, 109.2, 110.6, 110.7, 110.8, 120.8, 122.5, 127.2, 128.2, 128.5, 128.7, 134.3, 136.0, 148.6, 148.8, 152.1, 152.6, 165.7, 174.2.

15f (67.5 MHz, $CDCl_3$): δ = 28.2, 44.6, 55.6, 56.1, 71.0, 103.4, 105.8, 111.4, 111.5, 120.7, 127.2, 128.2, 128.8, 134.5, 136.0, 138.7, 148.8, 152.7, 160.4, 165.5, 174.5.

15g (67.5 MHz, $CDCl_3$): δ = 28.2, 45.0, 56.2, 56.3, 61.0, 71.0, 105.7, 111.4, 111.5, 120.7, 127.3, 128.4, 128.9, 131.8, 134.4, 136.1, 141.1, 148.9, 152.8, 152.9, 165.6, 174.4.

16a (67.5 MHz, DMSO-d_6): δ = 27.8, 43.5, 56.1, 111.7, 111.9, 114.5, 119.2, 120.8, 128.2, 128.5, 131.4, 136.1, 147.5, 152.6, 155.8, 164.7, 170.3.

16b (67.5 MHz, DMSO-d_6): δ = 27.7, 45.0, 55.8, 56.1, 111.7, 113.8, 114.6, 116.9, 118.9, 120.7, 129.7, 136.2, 138.8, 147.5, 152.7, 159.4, 165.4, 174.2.

16c (67.5 MHz, acetone-d_6): δ = 27.4, 44.5, 55.5, 111.2, 112.0, 113.9, 118.1, 119.4, 129.3, 131.5, 132.2, 133.9, 136.2, 139.0, 147.0, 152.1, 165.1, 172.0.

16d (67.5 MHz, DMSO-d_6): δ = 27.2, 44.8, 55.4, 55.6, 111.2, 113.3, 114.1, 118.6, 128.4, 130.7, 135.5, 146.9, 152.0, 162, 165.1, 173.6.

16e (100 MHz, DMSO-d_6): δ = 27.2, 44.9, 55.5, 55.6, 55.7, 110.6, 111.2, 111.6, 114.1, 118.6, 122.6, 128.5, 135.5, 147.0, 148.1, 151.8, 152.1, 165.1, 173.7.

16f (67.5 MHz, $CDCl_3$): δ = 28.1, 44.7, 55.6, 56.2), 103.3, 105.8, 111.0, 113.1, 120.1, 135.4, 138.8, 146.0, 150.8, 160.2, 165.5, 174.4.

16g (67.5 MHz, $CDCl_3$): δ = 28.0, 45.0, 56.2, 56.3, 61.0, 105.7, 111.0, 113.2, 120.2, 131.9, 135.4, 141.1, 146.1, 150.8, 152.9, 165.6, 174.4.

17a (100 MHz, DMSO-d_6): δ = 27.4, 43.4, 56.0, 56.1, 111.4, 112.4, 120.3, 122.0, 126.3, 127.2, 128.1, 131.2, 133.8, 142.7, 150.6, 155.4, 163.7, 169.9.

17b (100 MHz, DMSO-d_6): δ = 26.8, 44.4, 55.3, 56.0, 112.4, 113.4, 116.7, 120.3, 122.1, 126.0, 129.3, 134.0, 137.8, 142.9, 150.6, 158.9, 164.3, 173.7.

17c (67.5 MHz, acetone-d_6): δ = 27.0, 44.5, 55.7, 112.1, 112.7, 118.0, 123.0, 126.8, 129.4, 131.6, 132.3, 134.2, 134.3, 138.5, 143.4, 151.4, 165.1, 172.0.

17d (67.5 MHz, $CDCl_3$/methanol-d_4 5:1): δ = 28.9, 46.5, 56.9, 57.7, 114.8, 114.9, 115.0, 124.3, 128.5, 129.2, 132.5, 135.1, 144.5, 152.5, 164.4, 166.7, 176.5.

17e (67.5 MHz, acetone-d_6): δ = 27.9, 45.8, 56.1, 56.2, 56.4, 111.2, 113.0, 113.3, 123.7, 123.8, 128.0, 129.3, 134.7, 143.8, 149.6, 152.1, 153.6, 165.4, 174.6.

17f (67.5 MHz, $CDCl_3$/methanol-d_4 10:1): δ = 27.6, 44.6, 55.5, 56.4, 103.7, 105.9, 113.1, 122.9, 127.0, 133.7, 138.1, 143.1, 150.9, 160.6, 165.0, 174.4.

17g (67.5 MHz, DMSO-d_6): δ = 27.2, 45.2, 56.5, 56.7, 56.8, 106.4, 112.9, 122.5, 126.7, 132.3, 134.4, 140.9, 143.3, 151.1, 153.0, 164.7, 174.1.

Elemental Analyses for selected compounds.

			**Found**			**Expected**	
**Compound**	**Formula**	**C**	**H**	**N**	**C**	**H**	**N**
**7a**	$C_{26}H_{27}NO_5$	71.9	6.27	3.16	72.04	6.28	3.23
**7b2**	$C_{26}H_{27}NO_5$	71.7	6.26	3.25	72.04	6.28	3.23
**7d**	$C_{26}H_{27}NO_5$	72.2	6.25	3.22	72.04	6.28	3.23
**7e**	$C_{27}H_{29}NO_6$	69.8	6.37	3.06	69.96	6.31	3.02
**8a**	$C_{18}H_{19}NO_4$	68.6	6.10	4.30	68.99	6.11	4.47
**8b**	$C_{18}H_{19}NO_4$	68.9	6.28	4.28	68.99	6.11	4.47
**8c**	$C_{18}H_{19}NO_4$	68.5	6.17	4.45	68.99	6.11	4.47
**8d**	$C_{19}H_{21}NO_5$	65.0	6.16	3.60	66.46	6.16	4.08
**8e**	$C_{20}H_{23}NO_6$	63.9	6.16	3.60	64.33	6.21	3.75
**9a**	$C_{18}H_{20}N_2O_6S$	54.8	5.38	7.50	55.09	5.14	7.14
**9b**	$C_{18}H_{20}N_2O_6S$	54.8	5.21	6.99	55.09	5.14	7.14

**9c**	$C_{18}H_{20}N_2O_6S$	54.7	5.12	6.99	55.09	5.14	7.14
**9d**	$C_{19}H_{22}N_2O_7S$	53.1	5.33	6.32	54.02	5.25	6.63
**9e**	$C_{20}H_{24}N_2O_8S$	52.9	5.35	6.04	53.09	5.35	6.19
**10a**	$C_{24}H_{23}NO_6S$	63.5	5.11	3.20	63.56	5.11	3.09
**11a**	$C_{17}H_{17}NO_6S$	55.7	4.68	3.85	56.19	4.72	3.85
**11b**	$C_{17}H_{17}NO_6S$	56.0	4.80	3.67	56.19	4.72	3.85
**11d**	$C_{16}H_{14}ClNO_5S$	52.0	4.02	3.66	52.25	3.84	3.81
**11e**	$C_{18}H_{17}NO_7S$	55.3	3.42	4.42	55.24	4.38	3.58
**12a**	$C_{17}H_{18}N_2O_8S_2$	45.8	4.04	6.20	46.15	4.10	6.33
**12c**	$C_{17}H_{18}N_2O_8S_2$	45.8	4.08	6.21	46.15	4.10	6.33
**12d**	$C_{16}H_{15}ClN_2O_7S_2$	42.9	3.43	6.20	43.0	3.38	6.27
**12e**	$C_{18}H_{18}N_2O_9S_2$	46.0	3.93	5.87	45.95	3.86	5.95
**14a**	$C_{25}H_{25}NO_4$	73.8	6.41	3.29	74.42	6.25	3.47
**14b**	$C_{25}H_{25}NO_4$	74.1	6.36	3.46	74.42	6.25	3.47
**14d**	$C_{25}H_{25}NO_4$	74.1	6.45	3.53	74.42	6.25	3.47
**14e**	$C_{26}H_{27}NO_5$	72.2	6.32	3.06	72.04	6.28	3.23
**14f**	$C_{26}H_{27}NO_5$	71.7	6.13	3.36	72.02	6.28	3.23
**14g**	$C_{27}H_{29}NO_6$	69.5	6.14	3.01	69.96	6.31	3.02
**15a**	$C_{25}H_{23}NO_5$	71.6	5.55	3.35	71.93	5.55	3.36
**15b**	$C_{25}H_{23}NO_5$	71.6	5.53	3.34	71.93	5.55	3.36
**15c**	$C_{25}H_{20}N_2O_4$ $0.25H_2O$	71.7	4.84	6.71	72.02	4.96	6.72
**15d**	$C_{25}H_{23}NO_5$	71.7	5.54	3.26	71.93	5.55	3.36
**15e**	$C_{26}H_{25}NO_6$	69.7	5.61	3.09	69.79	5.63	3.13
**15f**	$C_{26}H_{25}NO_6$	69.4	5.64	3.18	69.79	5.63	3.13
**15g**	$C_{27}H_{27}NO_7$	67.5	5.73	2.91	67.91	5.70	2.93
**16a**	$C_{18}H_{17}NO_5$ $0.5H_2O$	64.3	5.08	4.17	64.28	5.39	4.16
**16b**	$C_{18}H_{17}NO_5$	65.8	5.20	4.21	66.05	5.23	4.28
**16c**	$C_{18}H_{14}N_2O_4$	66.5	4.55	8.39	67.07	4.38	8.69
**16d**	$C_{18}H_{17}NO_5$	66.2	5.25	4.14	66.05	5.23	4.28
**16e**	$C_{19}H_{19}NO_6$	63.6	5.34	3.98	63.86	5.36	3.92
**16f**	$C_{19}H_{19}NO_6$	63.8	5.37	3.90	63.86	5.36	3.92
**16g**	$C_{20}H_{21}NO_7$ $0.5H_2O$	60.4	5.58	3.49	60.60	5.59	3.53
**17a**	$C_{18}H_{18}N_2O_7S$	52.6	4.54	6.70	53.20	4.46	6.89
**17b**	$C_{18}H_{18}N_2O_7S$	53.2	4.53	6.66	53.20	4.46	6.89
**17c**	$C_{18}H_{15}N_3O_6S$	53.4	3.83	10.1	53.86	3.77	10.47
**17d**	$C_{18}H_{18}N_2O_7S$	53.1	4.41	6.85	53.20	4.46	6.89
**17e**	$C_{19}H_{20}N_2O_8S$	52.1	4.62	6.36	52.29	4.62	6.42
**17f**	$C_{19}H_{20}N_2O_8S$	52.3	4.68	6.32	52.29	4.62	6.42

**17g**	$C_{20}H_{22}N_2O_9S$	52.0	4.92	5.57	51.50	4.75	6.01
**18a**	$C_{19}H_{21}NO_5$	66.8	6.10	4.00	66.46	6.16	4.08
**18b**	$C_{19}H_{21}NO_5$	66.3	6.39	4.03	66.46	6.16	4.08
**19a**	$C_{19}H_{22}N_2O_7S$	54.4	5.43	6.38	54.02	5.25	6.63
**19b**	$C_{19}H_{22}N_2O_7S$	53.7	5.49	6.50	54.02	5.25	6.63

Computational Methods.

The Schrödinger software running under Maestro version 9.2.112 was used for all computational work. The crystal structure of **17f** has been solved. One molecule was taken from this structure and run through a brief geometry optimisation procedure. This resulted in a slight change in the dihedral angle A-B-C-D from 31.6º to 29.0º and in the dihedral angle B-C-D-E from 48.1º to 47.2º (Figure 1).

Figure 1. Dihedral angles ABCD and BCDE in **17f**.

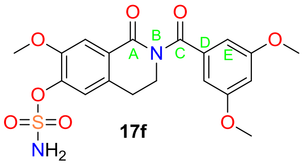

17f

The Current Energy protocol within the MacroModel program was used to calculate the energy in the compound.

Total Energy =	-224.1113 kJ/mol
Stretch =	13.3092 kJ/mol
Bend =	42.5224 kJ/mol
Torsion =	80.0845 kJ/mol
Improper Torsion =	6.7536 kJ/mol
VDW =	89.2127 kJ/mol
Electrostatic =	-344.3110 kJ/mol
Explicit Hydrogen Bonds =	0.0000 kJ/mol
Cross Terms =	0.0000 kJ/mol
Solvation =	-111.6828 kJ/mol

Both dihedral angles were manually and independently adjusted in steps of 30º and the energy of the conformers was calculated as above. **16g** and **17g** were built by altering the crystal structure of **17f** to yield the desired structure which was then run through a brief geometry optimisation procedure. Conformers were generated and molecular energies calculated as described above.

Table 1. Conformer Energies for **17f**. [a]Data in kJ/mol.

**Dihedral A-B-C-D**	**Dihedral B-C-D-E**					
	**17.2**	**47.2**	**77.2**	**107.2**	**137.2**	**167.2**
**29.0**	-153.8	-224.1	-215.9	-187.1	7098.6	23188.6
**59.0**	-202.3	-204.3	-192.5	-178.7	-30.8	-157.6

**89.0**	-179.4	-177.3	-165.3	-165.3	-174.4	-176.7
**119.0**	-165.7	-66.7	-156.7	-166.6	-179.4	-180.8
**149.0**	2743.1	5097.0	-165.4	-183.4	-195.4	-139.4
**179.0**	3118.5	-86.8	-185.3	-187.5	-109.5	3146.5
**209.0**	-71.2	-200.9	-192.6	-171.8	11999.6	3011.5
**239.0**	-181.4	-188.7	-177.1	-159.9	178.7	0.013
**269.0**	-173.6	-171.1	-162.1	-162.4	-168.7	-168.6
**299.0**	-107.4	300.1	-159.4	-176.2	-188.2	-183.6
**329.0**	159239.8	2400.1	-178.4	-200.0	-203.7	87.4
**359.0**	34002.1	-133.4	-208.0	-204.5	48.8	20604.9
